# Particulate and drug-induced toxicity assessed in novel quadruple cell human primary hepatic disease models of steatosis and pre-fibrotic NASH

**DOI:** 10.1007/s00204-021-03181-2

**Published:** 2021-10-20

**Authors:** Ali Kermanizadeh, Jessica Valli, Katarzyna Sanchez, Simon Hutter, Agnieszka Pawlowska, Graeme Whyte, Wolfgang Moritz, Vicki Stone

**Affiliations:** 1 Human Sciences Research Centre, University of Derby, Derby, UK; 2 School of Engineering and Physical Sciences, Heriot Watt University, Edinburgh, UK; 3 InSphero AG, Wagistrasse 27a, Schlieren, Switzerland

**Keywords:** 3D primary human quadruple-cell liver microtissue model, Steatosis, NASH, In vitro hepatotoxicity, Pre-existing disease state, In vitro vs. in vivo relevance

## Abstract

In an effort to replace, reduce and refine animal experimentation, there is an unmet need to advance current in vitro models that offer features with physiological relevance and enhanced predictivity of in vivo toxicological output. Hepatic toxicology is key following chemical, drug and nanomaterials (NMs) exposure, as the liver is vital in metabolic detoxification of chemicals as well as being a major site of xenobiotic accumulation (i.e., low solubility particulates). With the ever-increasing production of NMs, there is a necessity to evaluate the probability of consequential adverse effects, not only in health but also in clinically asymptomatic liver, as part of risk stratification strategies. In this study, two unique disease initiation and maintenance protocols were developed and utilised to mimic steatosis and pre-fibrotic NASH in scaffold-free 3D liver microtissues (MT) composed of primary human hepatocytes, hepatic stellate cells, Kupffer cells and sinusoidal endothelial cells. The characterized diseased MT were utilized for the toxicological assessment of a panel of xenobiotics. Highlights from the study included: 1. Clear experimental evidence for the pre-existing liver disease is important in the augmentation of xenobiotic-induced hepatotoxicity and 2. NMs are able to activate stellate cells. The data demonstrated that pre-existing disease is vital in the intensification of xenobiotic-induced liver damage. Therefore, it is imperative that all stages of the wide spectrum of liver disease are incorporated in risk assessment strategies. This is of significant consequence, as a substantial number of the general population suffer from sub-clinical liver injury without any apparent or diagnosed manifestations.

**Supplementary Information:**

The online version contains supplementary material available at 10.1007/s00204-021-03181-2.

## Introduction

The expanded commercial utilisation of engineered nanomaterials (NMs), which undoubtedly leads to increased exposure to users and consumers, highlighting the necessity to better evaluate the potential safety of these substances (Johnston et al. 2012; Kermanizadeh et al. 2015; 2018; Vance et al. 2015). Generally, toxicological studies are conducted using healthy test models that do not represent the responses of vulnerable individuals within the populations. Additionally, many investigations lack repeated exposures to low concentrations of NMs that better represent real-life exposures. Inhalation and ingestion studies suggest that approximately 1% of the NM dose becomes systemic (reviewed in Kermanizadeh et al. 2015). In addition to occupational and consumer NM exposure, which is inhaled and ingested, a number of nanomedicines have been developed for intravenous administration, resulting in intentional direct entry of materials into the blood (Balasubramanian et al. 2010; Kermanizadeh et al. 2018).

The liver is the body’s principal detoxification centre, removing foreign substances and waste products (Kmeic 2010). The organ is composed of very distinct populations of cells which function cohesively to perform the organ functions in health and disease. The liver is the principal site of drug metabolism. From the toxicological perspective, numerous drug metabolites are pharmacologically active and often more harmful than the parent compound. Drug-induced liver injury (DILI) remains a major obstacle in drug development due to either preclinical toxicity or being pulled from the market due to over adverse effects in patients once approved (Kuna et al. 2018). The combined presence of a potent innate immune response along with the tendency for the hepatic cells to be routinely exposed to xenobiotics can result in substantial liver inflammation after exposure to hepatotoxins. Additionally, the organ is understood to be a major storage organ for non-soluble xenobiotics (Lee et al. 2013; Lipka et al. 2010; Modrzynska et al. 2018) as exemplified in a recent study in which NM aggregates were detectable in the liver sinusoids (often in the KCs) up to 180 days post exposure (Modrzynska et al. 2018).

Non-alcoholic fatty liver disease (NAFLD) is the hepatic manifestation of metabolic syndrome and refers to a wide spectrum of liver disorders that can be broadly classified into two main categories: non-alcoholic fatty liver disease (steatosis) and non-alcoholic steatohepatitis (NASH), with the latter recognised as the progressive form of NAFLD. Globally, NAFLD affects approximately 25% of the overall population, among whom nearly up to 30% progress to NASH (Younossi et al. 2018). The present understanding of the progression of liver disease is underpinned by pro-inflammatory changes in the organ. Chronic inflammation and subsequent repair can ultimately lead to severe damage manifested as fibrosis which can progress to cirrhosis and potentially even to hepatocellular carcinoma (HCC) (Ioannou et al. 2020; Safari et al. 2019). Although the processes that determine fat accumulation in the hepatocytes are principally clear, the mechanisms associated with the progression of the disease are not yet fully understood. Due to the role of inflammation in the progression of liver disease and in the inflammatory responses of many tissues to NMs (Kermanizadeh et al. 2014; Li et al. 2020; Ma et al. 2020), there is potential for NAFLD diseased patients to exhibit different responses to NMs compared to healthy individuals.

This study utilises a scaffold-free 3D liver microtissue (MT) spheroid model which incorporated all the relevant primary human liver cell types responsible for the development of the hepatic disease—hepatocytes, hepatic stellate cells, Kupffer cells (KCs) (resident liver macrophages) and sinusoidal endothelial cells. Here, we designed and validated two novel in vitro disease models which represent steatotic and pre-fibrotic NASH liver, both benign medical conditions which often show no clinical symptoms but are highly prevalent in the general population and often underreported (Blais et al. 2015; Harris et al. 2017). The diseased MT were then used for toxicological assessment of a variety of engineered NMs (selected from the panel of materials selected in H2020 funded PATROLS project) and a well-known hepatotoxic drug (Isoniazid) in the context of a non-symptomatic, affected liver. This series of experiments benefited from long-term very low dosing, as well as the incorporation of recovery periods, in an attempt to account for the organ’s recovery capacity in vivo. Furthermore, a novel tissue clearance protocol for liver MT is introduced which allowed for improved visualisation of tissue layers without the necessity for sectioning allowing for a better understanding of the internalisation, interaction and penetration of materials in the cells within the spheroid. Finally, for the very first time we investigated stellate cell activation following particulate exposure. The importance of this study is emphasised by in vivo data demonstrating models of pre-existing hepatic disease enhances NM-induced hepatotoxicity (Du et al. 2018; Kermanizadeh et al. 2017, 2020a).

## Materials and methods

### Liver MT maintenance and disease induction

The study utilized 3D InSight™ human liver microtissues (MTs) composed of multi-donor (pooled cells from 10 donors) primary human hepatocytes in co-culture with non-parenchymal cells (NPC), containing primary human Kupffer cells, endothelial cells, and stellate cells (hepatocyte lot IPHH_18, NPC lotIPHN_15 and IPHS_13) (InSphero AG, Switzerland). A total of 46 MT plates (96 wells/plate) were utilized in this study The healthy MTs were maintained in 3D InSight™ Human Liver Lean Maintenance Medium (CS-07-305B-01, InSphero) at 37 °C, 5% CO_2_, 95% humidity with the fresh medium added (70 μl per well) on the day of arrival.

**Fig. 1:**
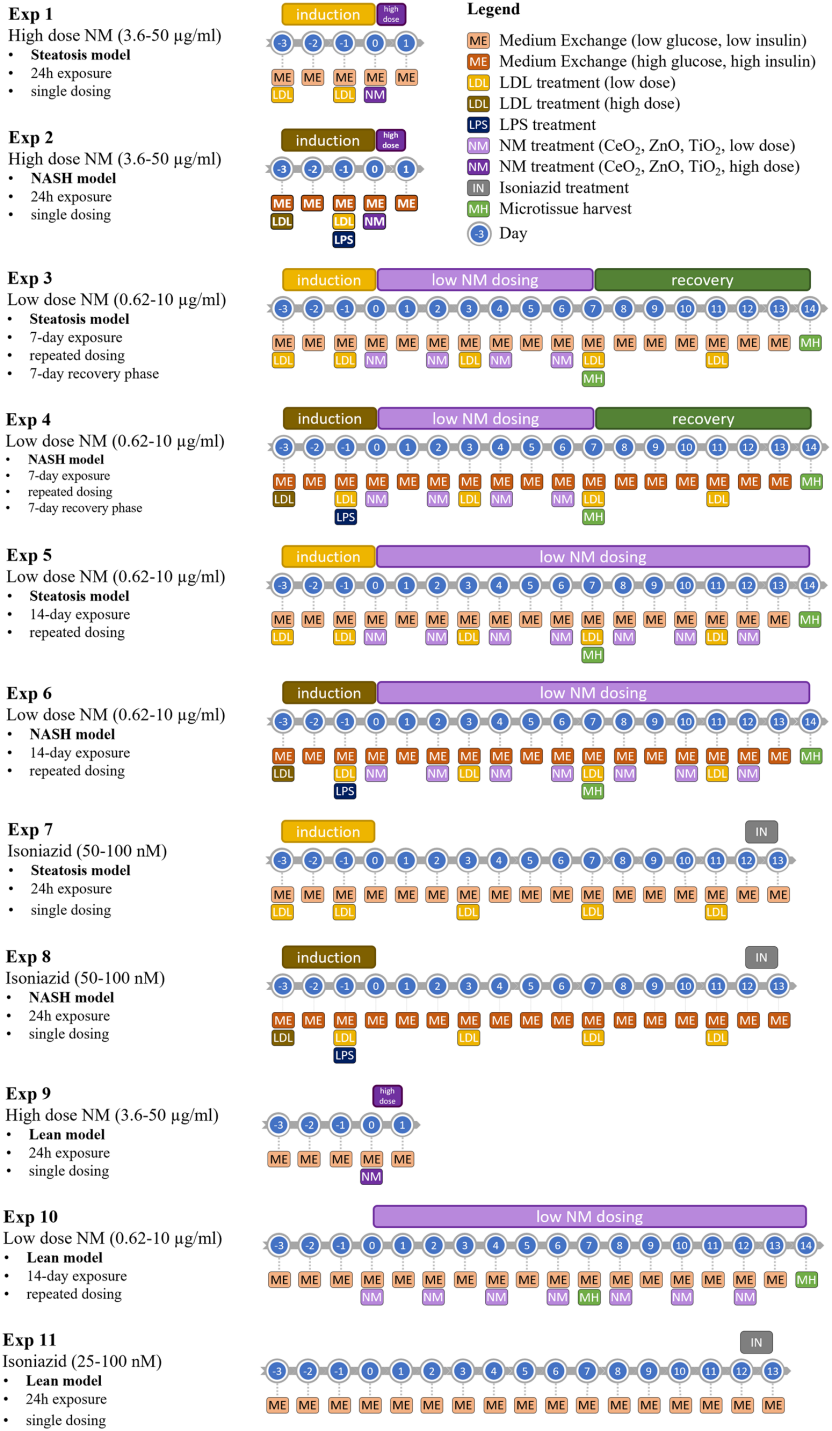
Disease state induction schemes and xenobiotic treatment regimens of the diseased MT over a period of 17 days

Induction of the diseased liver phenotypes, i.e., steatosis and pre-fibrotic NASH, started 3 days prior to NM exposure. Steatosis was induced and maintained with lean maintenance medium (3D InSight™ Human Liver Lean Maintenance Medium) containing normophysiological levels of glucose (5.6 mM) and insulin (0.1 nM), spiked for 24 h with low-dose (120 mg/dl total cholesterol) human plasma low-density lipoprotein (LDL) fraction (Lee Biosolutions, USA) on day -3, -1, 3, 7, and 11 (day 0 represents the first day of xenobiotic exposure) (Fig. 1). Medium conditions were maintained throughout the entire xenobiotic treatment and recovery period.

The pre-fibrotic NASH condition was induced with 3D InSight™ Human Liver NASH2.0 Basal Medium (CS-07–305-01; InSphero) containing supraphysiological carbohydrate (6.6 mM glucose, 10 mM fructose) and insulin (0.85 nM) levels supplemented by high-dose LDL (day -3, 200 mg/dl total cholesterol) and LPS (day -1, 5 μg/ml)). The medium was spiked with low-dose LDL (120 mg/dl total cholesterol) on days -1, 3, 7, and 11. The same medium conditions were applied throughout the entire NM treatment and recovery period.

### Liver MT characterisation

#### ATP content

Intracellular adenosine triphosphate (ATP) levels was determined using CellTiter-Glo^®^ Luminescent Cell Viability Assay 2.0 (Promega, USA), following a slightly modified protocol. In brief, PBS (w/o magnesium and calcium) was mixed with the CellTiter-Glo Reagent (1:1). Prior to addition of 40 μl of assay buffer mix to each well containing MTs, media supernatants were removed. MTs were incubated with assay buffer mix for 20 min in the dark while shaking on a horizontal plate shaker. After vigorous mixing in the culture plate, the whole lysate was transferred to a white assay plate (Greiner Bio-one, Austria) and luminescence was measured by a Tecan M200 Pro Infinite plate reader.

#### Triglyceride assay

Triglycerides were measured with Promega’s Triglyceride-Glo™ assay (J3160) according to the manufacturer’s protocol with slight modifications. In brief, MTs were washed in PBS (w/o magnesium and calcium), lysed in 25 µl lysis solution (including lipase) for 40 min on a rotational shaker. The lysate was then transferred into a white assay plate and 25 µl of detection reagent was added. After one hour on a rotational shaker luminescence was measured by a Tecan M200 Pro Infinite plate reader. Free glycerol in MTs has was not measured as detected quantities are neglectable.

#### Histology

Liver MTs were harvested on day 7 and 14, washed in PBS and fixated in 4% PFA for 1 h at room temperature. Fixed spheroids were embedded in Eppendorf tubes with 2% Agarose and further processed for paraffin embedding and sectioning of 3 µm thick sections. Standard haematoxylin and eosin (H&E), Masson’s Trichrome staining, and immunohistochemical staining for CD68 (NCL-L-CD68; Novocastra Laboratories Ltd., UK) were performed.

### RNA Sequencing and analysis

RNA Sequencing (RNA-Seq) was performed by BioClavis, LTD, using TempO-Seq^®^ technology (BioSpyder Technologies, Inc.). The Human Whole Transcriptome 2.0 assay, consisting of 22,537 probes targeting 19,701 genes, was utilized. Sample generation process was as follows: on days −3, 0, 3, 7 and 14 of treatment, single MTs were washed in PBS without Ca^2+^/Mg^2+^ and lysed in 15 ml of 1 × Enhanced Lysis Buffer (BioSpyder Technologies, Inc.). Lysates were sent to BioClavis for library generation and sequencing using the Illumina sequencing platform. After sample demultiplexing and obtaining single FASTQ files, reads alignment and counting were performed using TempO-SeqR data analysis platform (BioSpyder Technologies, Inc.). Probe-wise raw count table was collapsed toward gene-wise count table by summating counts for probes associated with the same gene. Low-expression genes (defined as genes for which number of samples with non-zero counts were less than 20% of all samples) were filtered out. To assess absolute gene expression, data were normalized as Transcripts per Million (TPM) counts. Principal Component Analysis and Differential Expression Analysis (DEA) were performed as implemented in *DESeq2* R package. Hierarchical clustering and heatmap generation were performed using Euclidean distance metric and ward. D2 linkage method was implemented in *pheatmap* R library. Pathway Analysis (PA) was performed using a competitive gene set test accounting for inter-gene correlation (Camera) as implemented in the *limma* R library. PA was run on MsigDB database version 7.4 selecting KEGG, Hallmark, PID, Reactome, BioCarta, Human Phenotype Ontology and WikiPathways sub-collections. For both, DEA and PA, Benjamini–Hochberg procedure for False Discovery Rate (FDR) was used to correct p values across contrasts. FDR cut-off was set to 0.001 and 0.05 for DEA and PA respectively. Furthermore, Surrogate Variable Analysis (SVA) was applied along DEA and PA to remove unintended batch effects using *sva* R package.

### Nanomaterials and isoniazid

The NMs were sourced as follows: ZnO (JRCNM01101a—NM 111, JRC Nanomaterials Repository—Italy), CeO_2_ (NM 212, JRC Nanomaterials Repository—Italy) and food-grade TiO_2_ (E171) (99.8% anatase and 0.2% rutile) was purchased online (www.bolsjehuset.dk). Isoniazid was purchased from Sigma, UK and used at 25, 50 and 100 mM for an exposure period of 24 h.

### Characterisation of NMs and xenobiotic treatment

A summarised list of the measured physical and chemical properties of the selected NMs has been re-produced from previous work (Kermanizadeh et al. 2013; 2019; JRC 2020). Furthermore, the hydrodynamic size distributions of the NMs dispersed in complete cell culture medium were determined at a concentration of 10 µg/ml by Dynamic Light Scattering (DLS) using a Zetasizer Nano-ZS (Malvern, USA). Next, a Pierce LAL Chromogenic Endotoxin Quantitation Kit (Thermo Scientific, UK) was utilized to test for possible endotoxin contaminations of the tested NMs according to the manufacturer’s guidelines.

NM dilutions were prepared following the NANOGENOTOX protocol (NANOGENOTOX 2019). The material and drug treatment regimens for acute or the repeated exposure experiments are summarised in Fig. 1.

The material and drug treatment regimens for acute or the two-week repeated exposure experiments (including recovery periods) are summarised in Fig. 1. To avoid potential complications with ageing of tissue between different plates, the three treatment repetitions were carried out on the same day (7–9 am, 11–13 am and 16–18 pm with a fresh batch of NMs prepared prior for each exposure). Finally, all concentrations of NMs are expressed as µg/ml. This decision is principled on the fact the liver cells are clustered in spheroids and expressing the treatments as µg/cm^2^ would not be appropriate. In addition, the wells are V-shaped, therefore the settling dynamics are likely to be different from a conventional well format.

### Adenylate kinase (AK) assay

The loss of cell membrane integrity was evaluated utilising a ToxiLight™ bioassay kit (Lonza, USA). Briefly, 20 µl of cell supernatant was transferred to a luminescence compatible plate before the addition of 80 µl of detection buffer. The plates were incubated for 5 min at room temperature and luminescence quantified. 24 h 0.1% triton (Sigma, UK) was utilised as the positive control.

### Cytokine secretion

The levels of human interleukin (IL)1ß, IL6, IL8, IL10 and tumour necrosis factor-α (TNF-α) secreted from the MT was determined in the cell supernatant using R&D Systems magnetic Luminex® Performance Assay multiplex kits (bead based immunoassay; Bio-techne, USA) according to the the manufacturer’s instructions. The protein concentrations were evaluated via a Bio-Rad^®^ Bio-Plex^®^ MAGPIX multiplex reader.

### Tissue homogenisation

A total of 15 MT were added to RIPA lysis buffer supplemented with a complete protease inhibitor mixture and a protein phosphatase inhibitor (Abcam, UK). For tissue homogenisation, pre-filled bead mill tubes (Thermo Fisher, UK) and an evolution homogeniser was used (Percellys, France). The samples were thoroughly mixed and stored on ice for 10 min before centrifugation at 10,000 g for 10 min. The supernatants were transferred to a fresh tube and centrifuged for further 10 min period. The protein concentrations were measured utilising a Coomassie Plus Bradford assay reagent (Thermo Scientific, UK). The supernatants were stored at − 80 °C.

### α-SMA ELISA

An increase in α-SMA isotype expressed by hepatic stellate cells reflects their activation to myofibroblast-like cell and is directly related to experimental liver fibrogenesis. In these experiments, the α-SMA levels from the supernatants of the homogenised control and treated MT tissue were quantified via a human α-SMA SimpleStep ELISA kit (Abcam, UK). The kit was utilised according to manufacturer’s instructions.

### Tissue clearance

Following the final exposure to the xenobiotics, the MTs were harvested washed with PBS, and fixed in 4% paraformaldehyde overnight at 4 °C. The MTs were washed 2 times with PBS. The MT was transferred directly into glass vials and sequentially incubated in 1 ml of each clearing solution, as described in Table 1 (all solvents purchased from Sigma UK). All the clearing steps were conducted in a fume hood at room temperature and in the dark.

**Table 1:** Main physical and chemical properties of investigated materials (adapted and reproduced from JRC nanomaterials repository 2020; Kermanizadeh et al. , 2013, 2019)

Material code	Material type	Phase	Primary size (nm)	Surface area [m^2^/g] (BET)	Known coating	Size in liver maintenance medium (DLS) (nm)^Ψ^
JRCNM01101a	ZnO	Coated	152	15	Triethoxy-caprylylsilane	692.2 ± 78.5
NM212	CeO_2_	Irregular and non-homogeneous	49	–	None	512.9 ± 8.2
E171,	Food grade TiO_2_	99.8% anatase and 0.2% rutile	–	–	None	614 ± 5.6

The hydrodynamic size distributions of the NMs dispersed in complete cell culture medium were determined at ^a^concentration of 10 µg/ml by Dynamic Light Scattering (DLS)

^Ψ^Size in biological media measured within 30 min of sonication

### Microscopy

The cleared exposed MTs in 100% dibasic ether were added to cavity microslides (Agar Scientific, UK), mounted with 25 mm diameter No. 1.5H high precision glass coverslips (Marienfeld Superio, UK) and sealed with Twinsil silicone (Picodent, Germany) components mixed in a 1:1 ratio. Excitation and emission spectra of samples were determined by performing Λλ scans on a Leica SP8 confocal laser scanning microscope with a Leica HC PL APO 20x/0.75 CS2 air objective. The samples were excited using a Supercontinuum White Light Laser at 5 nm intervals from 470 to 670 nm and were imaged in 10 nm-wide detection windows from 480 to 780 nm with a minimum gap of 10 nm between excitation and detection wavelengths. The excitation and emission maxima were calculated using the Excitation/Emission Contour Plot processing tool with bilinear interpolation in the Leica LAS X software. The imaging of samples was performed using a Leica SP5 confocal laser scanning microscope with a Leica HC PL APO 63 × /1.2 CORR CS2 water-immersion objective. The samples were excited using a Supercontinuum White Light Laser and detected using a Leica HyD hybrid detector (excitation and detection wavelengths specified in figures). Finally, the images were processed in FIJI Images containing both low and high-intensity structures were processed using a non-linear histogram adjustment to allow for the visualisation of lower intensity structures (gamma values specified in figures).

### Statistical analysis

Unless otherwise stated, the data are expressed as mean ± standard error of the mean (SEM). For statistical analysis, the experimental results were compared to their corresponding control values using full-factorial ANOVA with Tukey’s multiple comparison carried out utilizing Minitab 19. A *p* value of < 0.05 was considered to be significant. The experiments were repeated a minimum of three occasions. All authors had access to the study data and had reviewed and approved the final manuscript.

## Results

### Characterisation of diseased MT

**Fig. 2:**
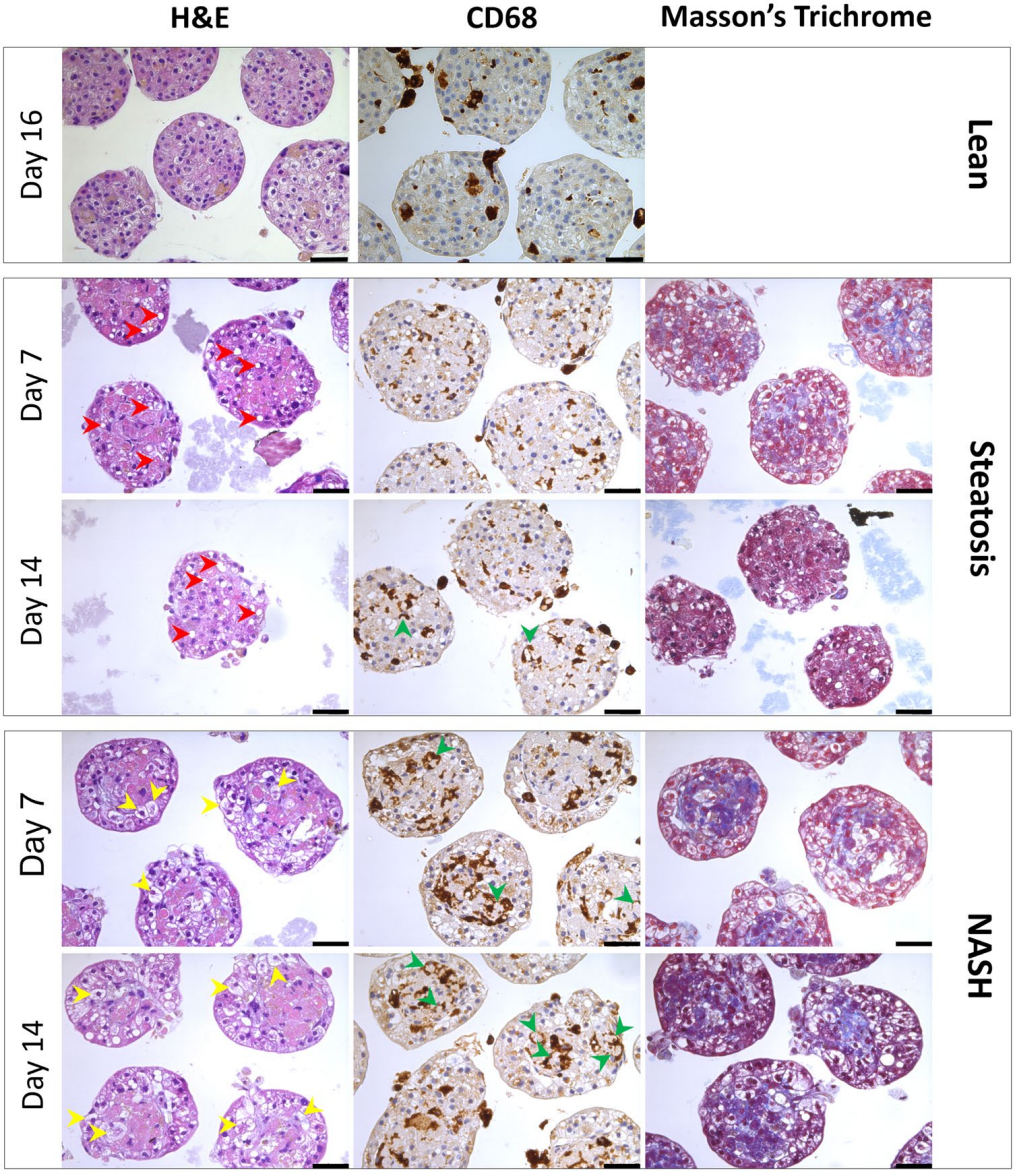
Histological and immunohistological staining demonstrating diseased liver phenotypes. Human liver MTs were collected at day 7 and day 14 after induction of steatosis and NASH. Macro-vesicular steatosis can be depicted by the presence of large white vacuoles representing large lipid droplets (red arrows). MTs treated for NASH induction revealed ballooning hepatocytes (yellow arrows) in addition to large lipid droplets. Additional morphological hallmarks associated with NASH were crown-like structures formed by CD68-postive macrophages (Kupffer cells, green arrows) and intensified Masson’s Trichrome staining of collagen deposition. Scale bar: 50 µm

For these experiments, the standard multi-cellular model (3D InSight™ Human Liver MT), was the starting point for the disease initiation models of steatosis and NASH induced by two different treatment schemes: a) steatosis—medium with supraphysiological glucose concentration or b) NASH—diabetic conditions at elevated sugar and insulin levels. Lipid loading occurred via supplementation of corresponding media with human plasma LDL fraction, on day -3, -1 and every 4th day thereafter. Activation of an inflammatory response was triggered by a single administration of LPS (5 μg/ml) on day -1 (Fig. 1). MT from both treatment groups were collected on day 7 and 14 for histological and biochemical analysis. The microtissues showed typical signs of lipid droplet inclusion by the presence of large vacuoles indicative of a macrovascular steatosis phenotype (Fig. 2). In the NASH condition a considerable number of enlarged hepatocytes with a white cytoplasm are observed, some of which reveal ballooning degeneration and Mallory bodies known as histopathological hallmarks of NASH (Supplementary Fig. 1). The same holds true for the presence of CD68-positive KCs, which are more numerous and larger in the NASH condition as compared to the steatotic situation. Interestingly, particularly in the NASH condition, agglomerates of CD68-positive cells form ring structures around degenerated hepatocytes, also known as hepatic crown-like structures (hCLS), which has been described as a histopathological feature in non-alcoholic steatohepatitis in mice and human (Itoh et al. 2013). Furthermore, Masson Trichrome staining was more pronounced in the NASH model, depicting unstructured collagen deposition indicative of a pre-fibrotic state (Fig. 2).

ATP levels in MTs treated for steatosis or NASH induction dropped by maximally 30% compared to the starting conditions right after the aggregation. While the ATP drop in lean conditions can be attributed to reduced energy stores (i.e., glycogen and triglycerides) as a consequence of low glucose and insulin concentrations. The ATP drop in steatotic and NASH MTs, which has also been observed in the steatotic liver of type 2 diabetic patients, suggests a metabolic stress response, either insulin resistance and/or mitochondrial disfunction which for steatosis stabilizes between day 7 and 14, but further progresses for the NASH conditions (Supplementary Fig. 2a). Lipid accumulation by LDL treatment can be demonstrated by measurement of intracellular triglyceride levels which compared to lean conditions increased by ~ 3–fourfold at day 7 and remains stable, with a slight decrease in the NASH conditions, again indicative of a metabolic stress situation (Supplementary Fig. 2a). To further progress steatosis into steatohepatitis, steatosis was induced with a high-dose LDL treatment on day -3, followed by an inflammatory LPS stimulus in combination with a low-dose LDL treatment at day -1 (Fig. 1). This resulted in a prolonged release of pro-inflammatory cytokine and chemokines, such as IL8, MIP-1α, MCP-1 (Supplementary Fig. 3). Of note, the steatosis induction without LPS stimulation led also to an increase of IL8 and MCP-1.

**Fig. 3:**
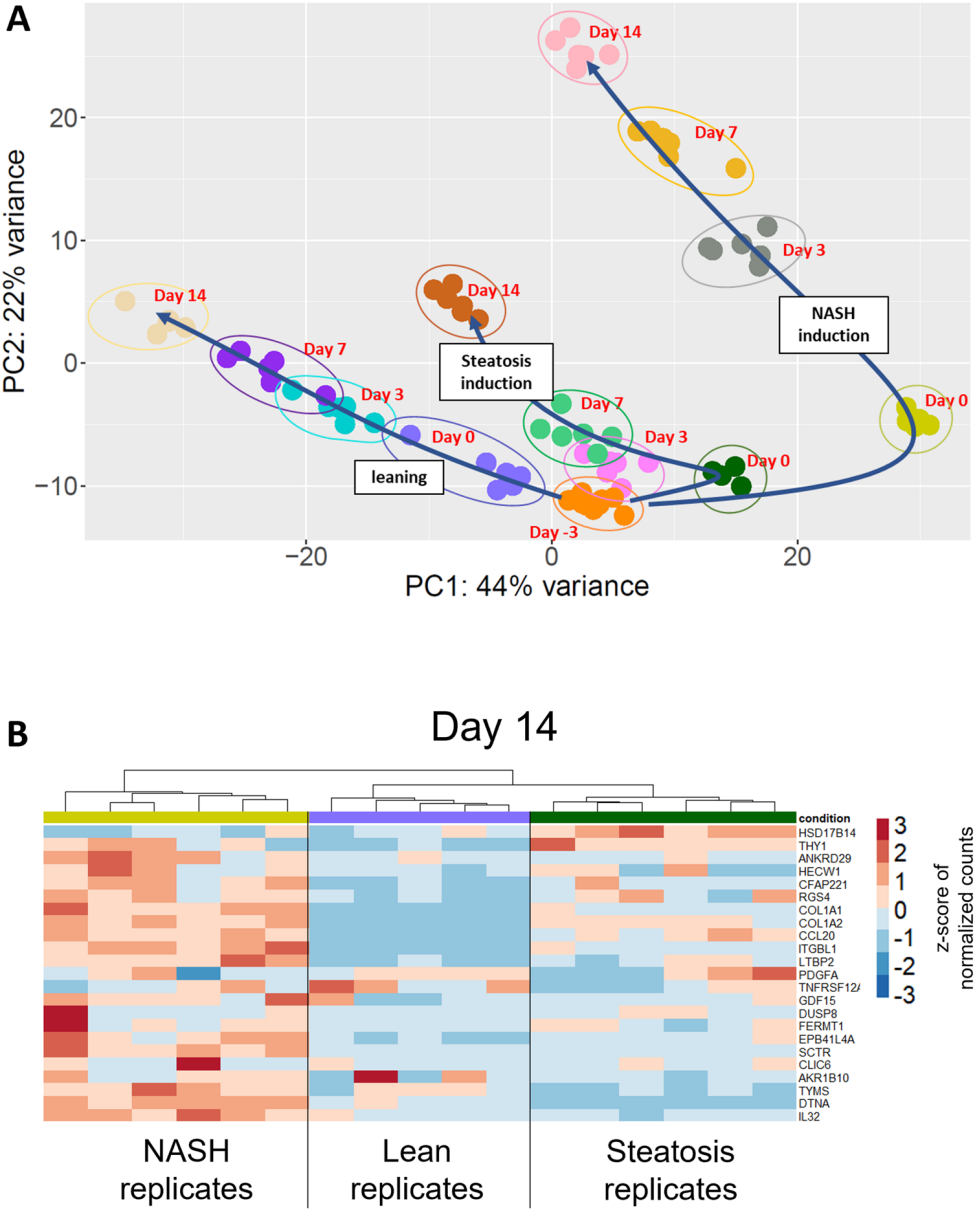
Transcriptome analysis of lean, steatotic and NASH model: **A** Time-resolved principal component analysis for changes in expression profile along with the different induction schemes for lean, steatotic and NASH phenotype starting form freshly aggregated human liver MTs on day -3. **B** Heatmap representing transcriptome data from lean, steatotic, and NASH samples applied on a 23-gene signature associated with the transition from non-alchoholic fatty liver to NASH. Values correspond to gene-wise z-score of normalized counts for upregulated (red) or downregulated (blue) genes. Progressive phenotype from lean to NASH is represented by the increasing number of genes with a positive z-score

To further confirm the establishment of three distinct metabolic phenotypes representing lean, steatotic and pre-fibrotic NASH disease models, whole transcriptome gene expression analysis was performed, based on a targeted approach using Biospyder’s TempoSeq sequencing platform. Starting from a standard liver microtissue at day -3 and applying different media with contrasting composition regarding carbohydrates (glucose, fructose), insulin and lipids (LDL), a principal component analysis (PCA) revealed three distinct lineages of clusters forming over a period of total 17 days (Fig. 3A). As expected, the lean model and NASH model represent the most distinct phenotypes with the steatotic model positioned in between. The gradual transformation into the respective steatotic or NASH phenotype over time is represented by a staggering number of differentially expressed genes when compared to the lean counterpart (Supplementary Fig. 4a). On day 14, out of 15,413 identified genes, 1379 and 2239 genes were differentially expressed in steatotic and NASH samples respectively, when compared to lean microtissues, nicely separated as two hierarchical clusters as presented by a heat map (Supplementary Fig. 4b). In both induction schemes, steatosis and NASH, the top 20 up-regulated pathways where primarily related to ECM remodelling while the top 20 down-regulated genes were associated with lipid-, cholesterol-, xenobiotic metabolism and oxidative phosphorylation (Supplementary Fig. 4c). The transcriptome profile of either model, i.e., lean, steatotic, and NASH, revealed the dynamics related to the main mechanisms responsible for NASH progression, namely altered lipid metabolism, inflammation and ECM remodelling (Supplementary Fig. 4d). Recent data has identified and validated a human liver-specific gene expression signature, composed of 25 transcripts which are associated with early stages of NAFLD and subsequent progression towards fibrosing NASH (Govaere et al. 2020). As expected, the NASH samples showed the highest correlation with the NASH progression-specific 25-gene set, where 20 out of 23 detectable transcripts were regulated in the same direction as described (19 up, 1 down), whereas the steatotic microtissues showed only the same behaviour in 3 out of 23 signatures genes (Fig. 3b). Differential expression (p_adj_ < 0.001) in relation to this 25-gene signature was established in 8 out of 23 for the steatotic model and in 10 out of 23 detectable transcripts in the NASH model (p_adj_ < 0.001, Supplementary Fig. 4e). The gene expression data support the notion that with the different induction schemes two discrete disease phenotypes could be obtained which represent distinct stages in the progression of NAFLD.

Altogether, these data clearly demonstrate that the two selected induction schemes are unmistakably able to generate distinguishable pathophysiological liver conditions, namely a moderate steatotic phenotype with a slightly inflamed state and a more progressed phenotype showing typical histopathological hallmarks of NASH but without pronounced fibrosis.

### Characterisation of pristine and dispersed NMs

An extensive list of the measured physiochemical properties for the materials as well as the hydrodynamic size distribution of the NMs in the liver MT maintenance medium is described in Table 2. In addition, no endotoxin contamination (≤ 0.25 EU/ml) was detected in any of the material suspensions.

**Table 2:** Time-line for toxicological end-points measured in the single or repeated exposure experiments over a period of 2 weeks

Experiment 1 and 2 and 9	End-points investigated
Day 1	AK assay, cytokine secretion, α-SMA
Experiment 3 and 4	End-points investigated
Day 1	AK assay, cytokine secretion
Day 3	AK assay,
Day 5	AK assay
Day 7	AK assay
Day 13	AK assay, cytokine secretion
Experiment 5, 6 and 10	End-points investigated
Day 1	AK assay, cytokine secretion
Day 3	AK assay,
Day 5	AK assay
Day 7	AK assay
Day 9	AK assay, cytokine secretion
Day 11	AK assay
Day 13	AK assay, cytokine secretion, α-SMA
Experiment 7, 8 and 11	End-points investigated
Day 13	AK assay, cytokine secretion

### Xenobiotic-induced cytotoxicity

**Fig. 4:**
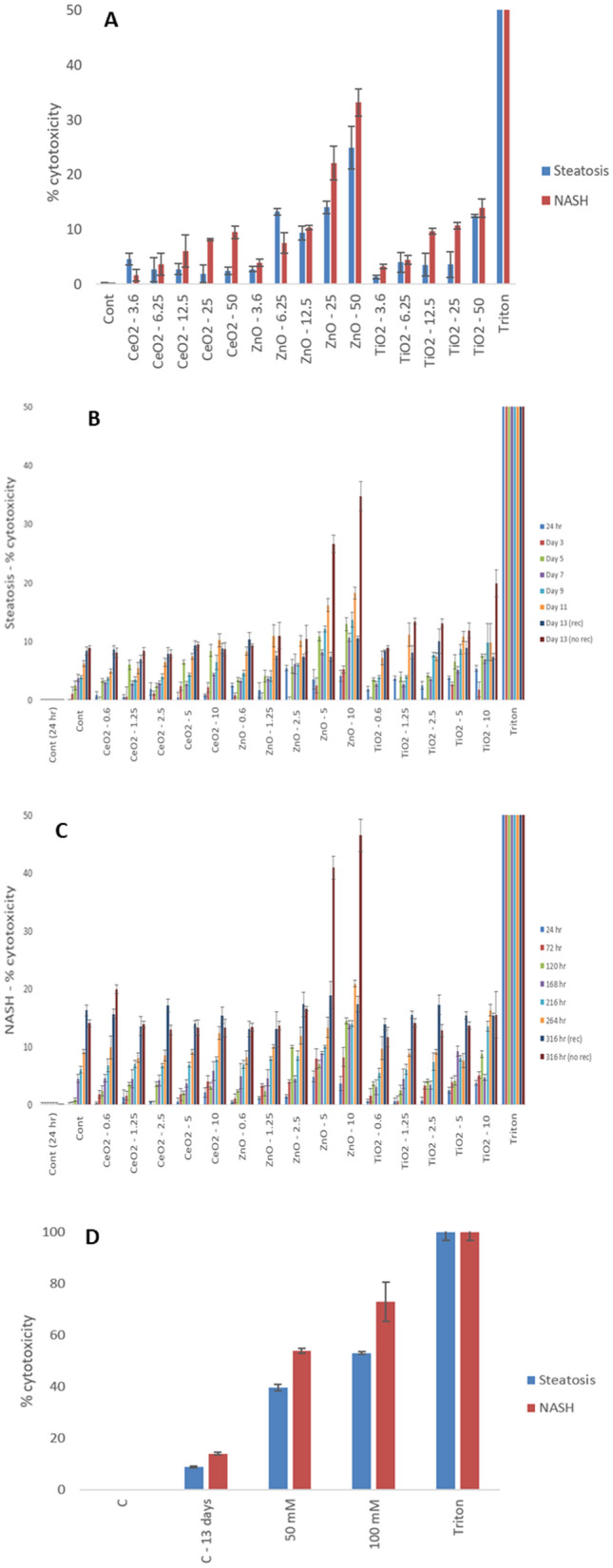
Cytotoxicity in the quadruple cells human liver MT following exposures to materials and Isoniazid for up 2 weeks as measured by AK release via ToxiLight™ cytotoxicity assay (material treatments µg/ml). **A** 24 h of material dosing of steatosis (Exp. 1) and NASH MT (Exp. 2), **B** 13 days of materials dosing of steatosis MT (Exp. 3 and 5), **C** 13 days of materials dosing of NASH MT (Exp. 4 and 6), and **D** 24 h dosing of 50 or 100 mM of isoniazid in steatosis (Exp. 7), and NASH MT (Exp. 8)**.** 0.1% Triton utilised for 24 h acted as a positive control for these experiments. The values represent mean ± SEM (*n* = 3)

The time-line for metabolic phenotype induction, single or repeated dosing regimens and assessment of different end-points over a period of 2 weeks is highlighted in Table 3. The cytotoxicity data (Fig. 4 and Supplementary Fig. 5) showed a concentration and time-dependent decrease in cell membrane integrity following repeated exposure to the NMs. This was most evident following exposure to the ZnO NMs (repeated dosing of 10 µg/ml up to day 13—lean MT 19.91 ± 0.42% vs. steatosis MT 34.8 ± 2.5% vs. NASH MT 46.6 ± 2.81%). Overall, the data demonstrated the approach of repeated low dose (up to 10 µg/ml) long-term exposure strategy to be undoubtably the most physiologically relevant and “realistic” strategy for hepatic nanotoxicology. A significant ageing effect was observed within the two diseased MT models at 2 weeks of cell culture which was more apparent for the NASH model (Fig. 3b, c). Generally speaking, the recovery period in experiments 3 and 4 were not all that effective in terms of the specific end-point (i.e. cell death—not all that surprising as generally there is very little proliferation in primary human hepatocytes in vitro and the compromised health status of the MT in this study). However, there was some recovery effect following ZnO treatment at higher dosages. A final crucially important NM-specific observation from the cytotoxicity data was the significantly higher NM-induced cytotoxicity in the two disease models as compared to healthy MT at the same concentrations (as exemplified above for ZnO NMs). The 24 h exposure of the MT to increasing doses of Isoniazid (25–100 mM) revealed the NASH MT to be more susceptible to the hepatotoxin that the steatosis tissue (i.e., healthy MT 49.5 ± 4.1, steatosis MT 53.1 ± 0.6% vs. NASH MT 72.9 ± 7.6) (Fig. 4d, Supplementary Fig. 5c).

**Table 3:** Tissue clearance protocol for primary human liver MT

Reagent	Incubation time
50% tetrahydrofuran (THF) (vol/vol)	25 min
70% tetrahydrofuran (THF) (vol/vol)	25 min
80% tetrahydrofuran (THF) (vol/vol)	25 min
100% tetrahydrofuran (THF) (vol/vol)	3 × 25 min
100% dichloromethane	20 min
100% dibasic ether	15 min

These findings demonstrate that incorporation and consideration of pre-existing disease states of the liver are very important not only in the augmentation of NM-induced adverse effects but in all reality also applies to hepatotoxicity induced by pharmaceuticals and chemicals.

### Cytokine secretion from liver MT following xenobiotic exposure

**Fig. 5:**
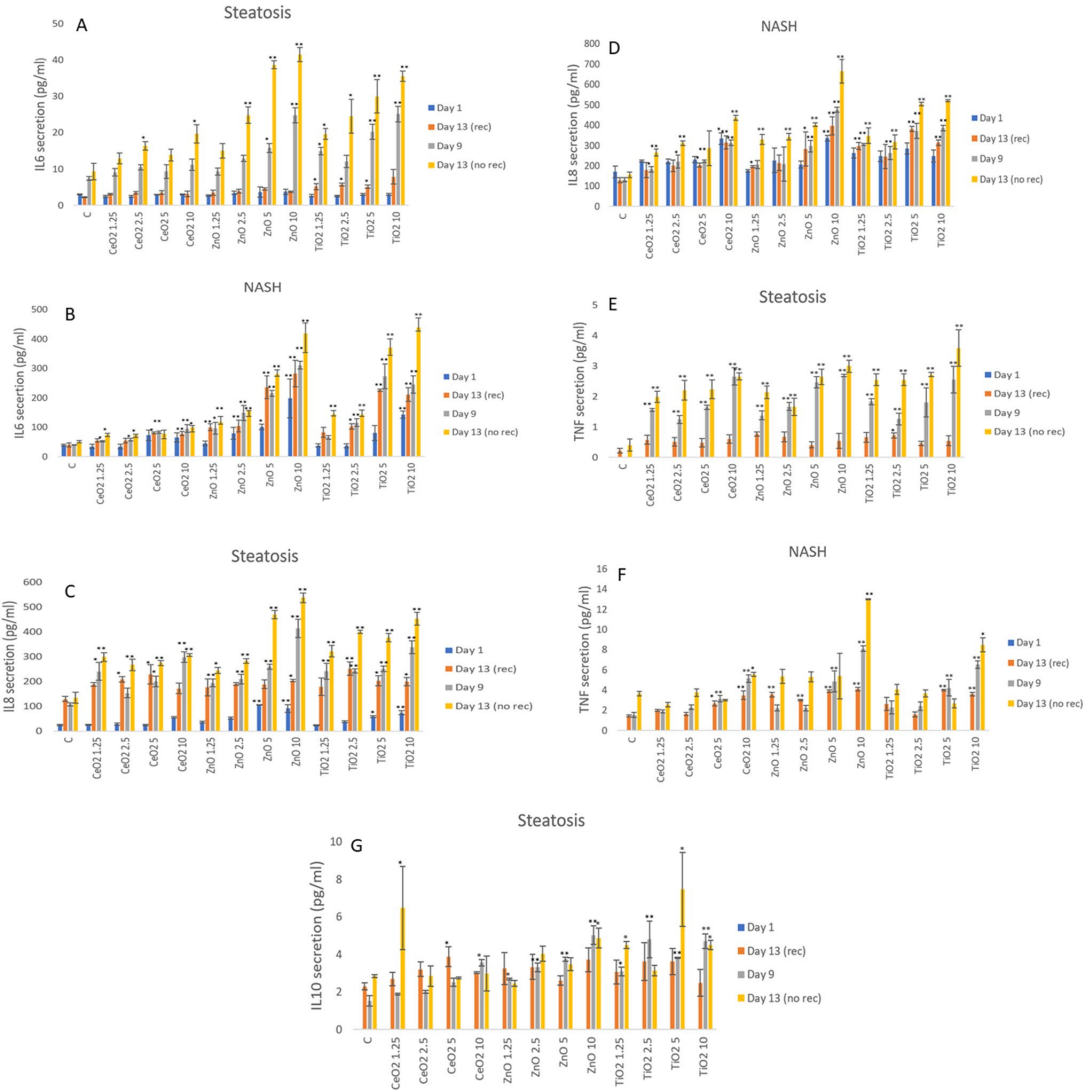
IL6, IL8, TNF-α, and IL10 levels from NM-exposed human liver MT. The tissues were exposed to cell medium alone (C) or repeated concentrations of NMs for up to 2 weeks – (**A**) IL6—steatosis, (**B**) IL6—NASH, (**C**) IL8—steatosis, (**D**) IL8—NASH, (E) TNF-α—steatosis, **F**) TNF-α—NASH, G) IL10—steatosis. The values represent mean ± SEM (*n* = 3) with significance indicated by *p < 0.05 and **p < 0.005 of NM-induced effects compared to a negative control. Treatment schemes with and without a recovery period where as illustrated in Fig. 1, Exp. 3 and 5 for steatosis and Exp. 4 and 6 for NASH

**Fig. 6:**
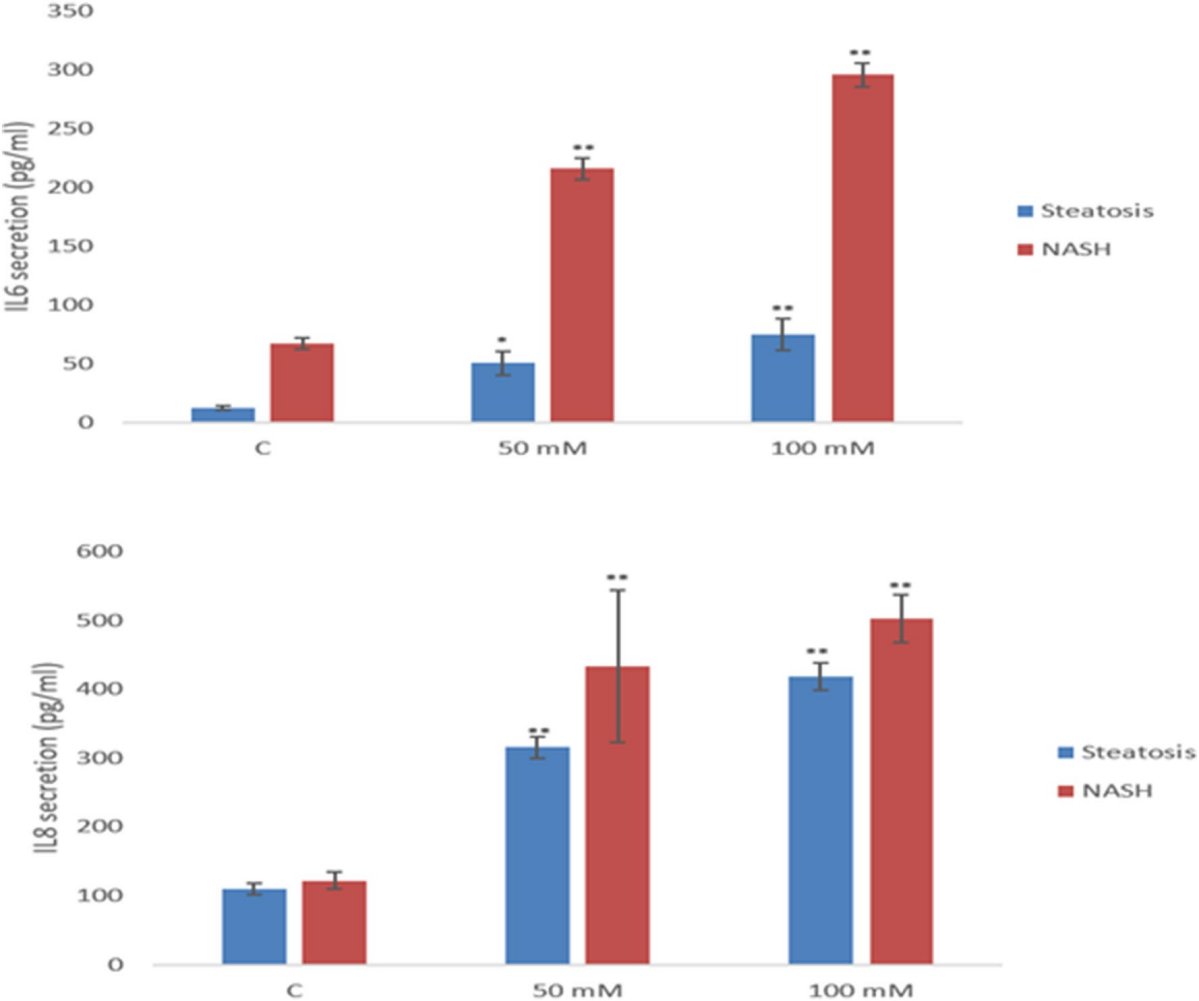
IL6 and IL8 secretion following a 24 h Isoniazid dosing of the two different diseased liver MT models at 50 or 100 mM (Exp 7 and 8). The values represent mean ± SEM (n = 3) with significance indicated by *p < 0.05 and **p < 0.005 of NM-induced effects compared to a negative control

The alterations in cytokine protein levels (IL1ß, IL6, IL8, IL10 and TNF-α) as a consequence of xenobiotic exposure was assessed within the supernatant of control, NM and drug-exposed liver MT (Fig. 5, 6 and Supplementary Fig. 6, 7 and 8). Firstly, no IL1ß was detected at any of exposure scenarios, disease states or time-points investigated. The high concentrations exposure data (Supplementary Fig. 6) revealed a concentration dependant increase in IL6 and IL8 secretion following exposure to all three material types. Interestingly at 24 h, there was an almost five-fold increase in IL8 (i.e. following exposure to ZnO NMs at 25 µg/ml—steatosis MT 83 ± 12.9 pg/ml vs. NASH MT 506.3 ± 9.82 pg/ml) and almost ten-fold increase in IL6 (i.e., following exposure to TiO_2_ NMs at 25 µg/ml—steatosis MT 11.84 ± 1.17 pg/ml vs. NASH MT 196.2 ± 13.5 pg/ml) in NM-induced cytokine levels in the NASH MT as compared to the steatosis MT. There was no detectable TNF-α or IL10 at 24 h following exposure to any of the materials at the concentrations administered.

Figure 5 shows a general trend of concentration-dependent increases in inflammatory cytokines over time with increased repeated dosing (most evident for ZnO and TiO_2_ NMs). From the data, two very important and notable observations were detected. First, there was a ten-fold increase in IL6 levels following NM exposure in the NASH MT as compared to steatosis MT (i.e., 13 day exposure of TiO_2_ NMs at 10 µg/ml—steatosis MT 35.49 ± 1.44 pg/ml vs. NASH 438.8 ± 33.1 pg/ml), and this was sustained across all timepoints (Fig. 5a, b). Similarly, but less pronounced, NM-induced IL8 and TNF-α levels were higher in the NASH model as compared to the milder disease MT (Fig. 5c, d, e, f). Second, there was no detectable IL10 (anti-inflammatory cytokine secretion) in any exposure or time points in the NASH liver MT yet an IL10 response was observed in the steatosis liver MT at the later time-point.

Interestingly in a similar pattern to above, a concentration-dependant increase in IL6 and IL8 was also observed in the diseased MT following exposure to isoniazid with the inflammatory response significantly higher for the NASH model compared to the steatosis tissue (i.e., 24 h 100 mM isoniazid exposure—IL6—healthy MT 25 ± 3.24 pg/ml, steatosis MT 74.4 ± 13.4 pg/ml vs. NASH MT 295.9 ± 10.1 pg/ml; IL8—healthy MT 240.9 ± 37.1, steatosis MT 418.5 ± 19.1 pg/ml vs. NASH MT 502.5 ± 34.9 pg/ml) (Fig. 6 and Supplementary Fig. 9).

Overall, the data clearly demonstrate the disease state is absolutely key in the governance of how the organ might deal with xenobiotic challenge, e.g., by modulation of an inflammatory response.

### Stellate cell activation following NM exposure

**Fig. 7:**
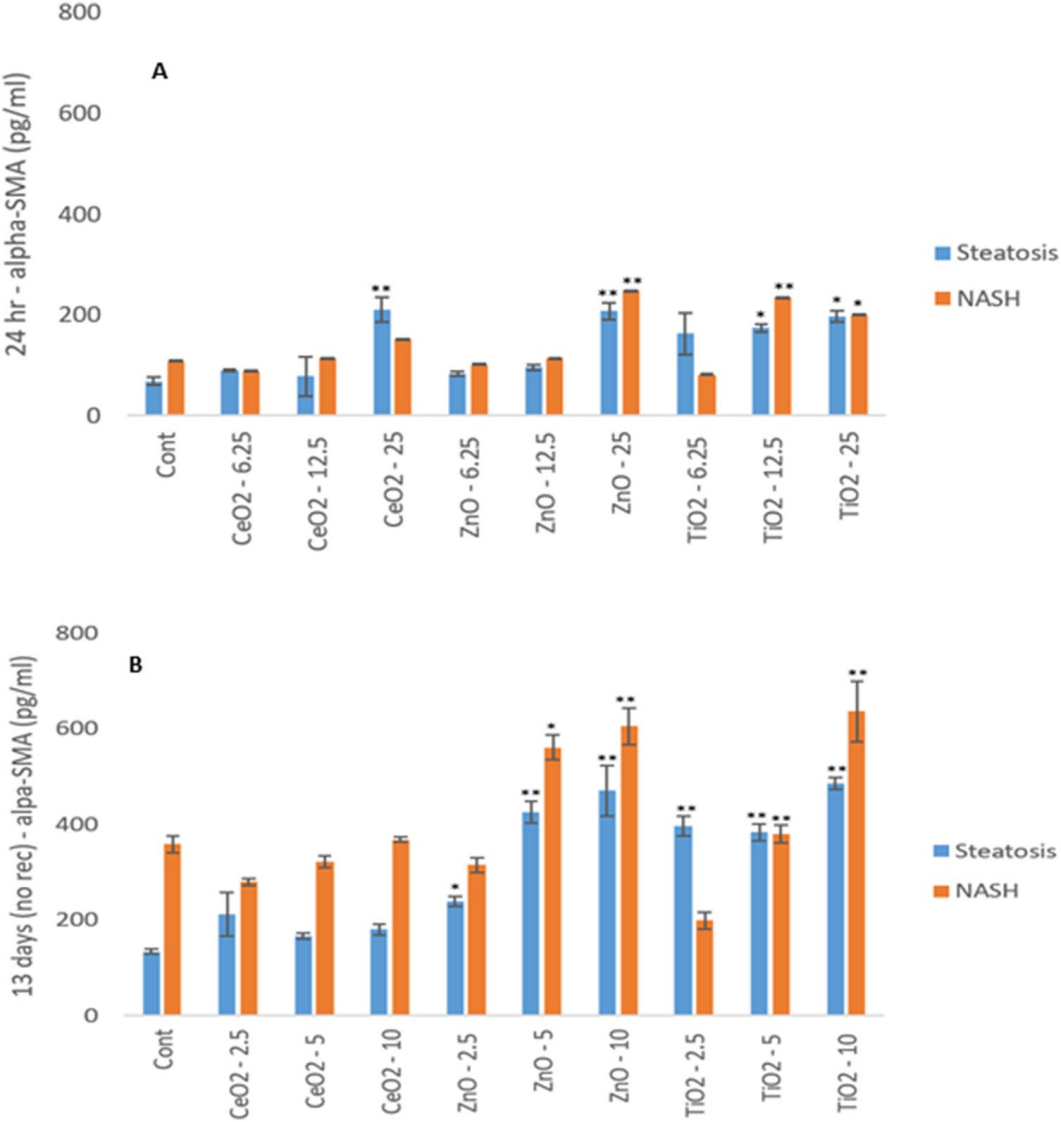
α-SMA protein quantification in the steatosis and NASH diseased MTs and following exposure to (**A**) a single dose of NMs for 24 h (Exp. 1 and 2), (**B**) repeated doses of NMs over a period of 2 weeks (Exp. 5 and 6). The values represent mean ± SEM (*n* = 3) with significance indicated by *p < 0.05 and **p < 0.005 of NM-induced effects compared to a negative control

α-SMA expression is considered a reliable marker of hepatic stellate cell activation and a key biomarker for liver fibrosis. In this study, as expected higher levels of α-SMA proteins were quantified in the NASH MTs over time as compared to the steatosis (Fig. 7 and Supplementary Fig. 10). For the very first time and crucially, the data demonstrated that specific NMs (i.e. ZnO and TiO_2_) were capable of activating stellate cells (most apparent for ZnO and TiO_2_ NMs following repeated dosing) (Fig. 7b).

### NM distribution in the MT

**Fig. 8:**
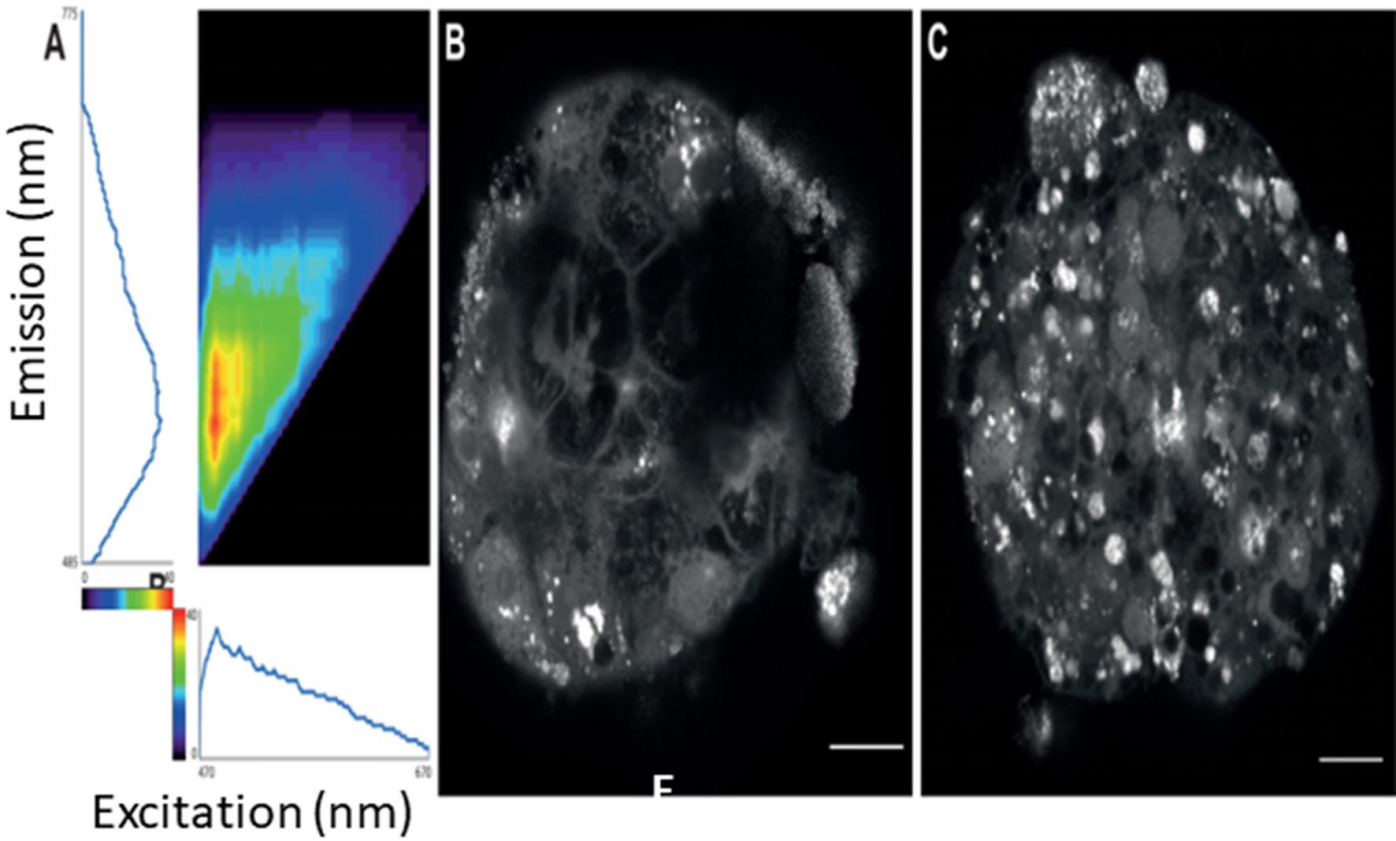
Detailed structures of cleared MTs visualised using autofluorescence alone. The autofluorescence of cleared MTs can be imaged at a broad range of excitation and detection settings: (**A**) excitation and emission spectra of autofluorescence of CeO_2_ NM nanomaterial-exposed MT. Maxima occur at Ex 485 nm/Em 560 nm, but the spectra are extremely broad allowing for imaging with various settings. Images (gamma = 0.7) show cleared cerium nanomaterial-exposed MT imaged with (**B**) excitation at 480 nm and detection from 490 to 795 nm, (**C**) excitation at 590 nm and detection from 600 to 795 nm. Scale bars − 20 μm

Due to the tightly packed three-dimensional structure of the spheroids, it was imperative to investigate whether the inner cells in the MT come in direct contact with low soluble NMs. For this purpose, we designed a novel tissue clearance protocol modified and specified for use for liver tissue (liver MT in the present study and rodents). The autofluorescence of cleared MTs allows for the identification of individual cells even in a dense mass as well as allowing for the visualisation of NM uptake and interaction with specific cells within the MT (Fig. 8). The tissue clearance enables imaging of layers (Supplementary video 1) throughout the MT which allows assessment of the localization and distribution of NM within tissue without the necessity for cross sections. A final and interesting finding was the NM interactions were not equal for every cell—in fact specific cells clearly interacted with NMs more extensively than others (suspected that these are the non-parenchymal cell populations as compared to the hepatocytes) (Fig. 8c).

## Discussion

The liver is a truly multi-functional organ with a variety of crucial roles for everyday life. Due to its large endogenous macrophage population and its ability for easy recruitment of circulating white blood cells, the liver is one of the primary organs responsible for innate immunity. Additionally and importantly to this investigation, the liver is the principal organ to exposure to gut-derived antigen and blood-sourced xenobiotics. In this novel study, particulate and drug-induced hepatotoxicity was assessed in quadruple cell human primary hepatic disease models of steatosis and pre-fibrotic NASH. The MT offers a metabolically active system in a 96 well plate format with no scaffolds or hydrogels that utilises primary human hepatic cells. The two diseased MTs are viable for periods of weeks, which allows for low-dose multiple xenobiotic treatments. This equates to more physiologically relevant and realistic exposure scenarios, making them useful for investigating hepatic toxicity and predicting such responses in vivo. In this study, it was intended to try and avoid NM-induced cell death and focus principally on sub-lethal effects, which is the only option available if physiological relevance is to be considered in the experimental design (discussed in detail in Kermanizadeh et al. 2020a). It is important to keep in mind that this was an in vitro experiment so naturally the concentration of NMs used are higher than a human might be exposed to via the inhalation or oral routes but the IV route of exposure cannot be ignored (this of great importance for the overall conclusions reached within these trials).

NAFLD is a multifactorial pathological condition affecting extremely large numbers globally (Benedict et al. 2017). The term NAFLD assembles a wide and varied spectrum of histological conditions, ranging from the simple hepatocyte free fatty acid accumulation (termed steatosis in this study), which can be further associated increasing severities of inflammation—NASH, necrosis and fibrosis. As with other causes of chronic liver disease, NAFLD increases the risk of hepatocellular carcinoma. In a recent theory described as “distinct hit” proposed pure fatty liver and NASH as two distinct conditions with visceral obesity and insulin resistance being the underlying causes (Alam et al. 2016), with a subsequent inflammatory injury on the organ resulting in the eventual development of NASH. In this study, the induction of NASH was achieved by treatment with LDL, LPS and sugars.

A growing body of literature has demonstrated that NM-induced adverse effects in the liver are significantly aggravated in the diseased organ (i.e. Du et al. 2018; Kermanizadeh et al. 2018; 2020a). Furthermore, the disease state of the liver might influence and impede the organ’s ability to regenerate and recover post-NM challenge. As an important and additional complication and as touched upon above, large numbers within the general population suffer from a wide spectrum of sub-clinical liver damage without any apparently visible disease manifestations. Therefore, it is critical that a range of liver diseases (mild to severe) are considered in hazard and risk assessment strategies. Aside from a healthy, lean phenotype, specific induction schemes allowed to create diseased in vitro liver models which are characteristic for two distinct stages of NAFLD, steatosis and pre-fibrotic NASH, which often remain clinically asymptomatic and undetected, yet occur at a high prevalence. Histological, metabolic and transcriptomic data revealed that the two disease models displayed pronounced features of typical hallmarks of early stage and progressing NAFLD, i.e., distorted lipid metabolism, inflammation and ECM re-modelling.

The cell membrane integrity data demonstrated a concentration and time dependent increase in cell death following repeated exposure to the NMs most notable following exposure to the ZnO NMs. Additionally, the 24 h exposure of the MT to increasing concentrations of Isoniazid revealed the NASH MT to be more susceptible to the hepatotoxin. One of the most interesting findings from the data is that the cell death induced by the particulate and chemical xenobiotics was significantly higher in the NASH model as compared to the steatosis MT at comparative concentrations. Moreover, the xenobiotic-induced cytotoxicity levels observed in the diseased MTs in this study (i.e., cell death following exposure to 7 doses of ZnO NM at 5 µg/ml steatosis MT 30% and NASH MT 40%) were substantially higher than cell death observed in the healthy multi-cellular primary exposed to the same particulates at the same dosing regimens. These findings are very valuable as they clearly demonstrate the increased susceptibility of the diseased model. It is therefore important to incorporate liver disease models in hazard assessment strategies and drug safety measurements. It is unlikely that the average adult in the general population in the western world will have a pristine liver as found in a young rodent, which is the most heavily relied upon test model in pre-clinical drug development safety assessments (Kuna et al. 2018). The same argument can be extended to question the rationale of data generation using in vitro liver test systems that are only representative of a healthy liver while ignoring the billions of individuals in general populace with potential undiagnosed liver conditions that can only be represented by the diversification of the toxicological testing strategies and test models utilised.

The inflammatory response in the absence of infection (sterile inflammation) has received considerable and increasing interest in hepatology in recent years. Sterile inflammation is a common outcome of a number of different liver diseases (including NASH). Sterile inflammation occurs when an organized inflammatory response occurs in the absence of any infection. If the sterile stimulus is not resolved, this drives chronic inflammation resulting in comprehensive tissue damage. It is believed that a sterile inflammatory response during liver injury is initiated by the breakdown of the hepatocyte membrane and release of their constituents (Woolbright et al. 2015, 2018). Sterile inflammation amplifies organ damage by the activation of inflammasomes. The recognition of PAMPs and DAMPs by pattern-recognition receptors expressed by leukocytes, in particular, leads to inflammasome activation resulting in activation of the protease caspase-1 and secretion of the inflammatory cytokines namely IL1-β and IL18 (Yazdani et al. 2017). Moreover, activation of TLRs on KCs also initiates processs responsible for activating the inflammasome. Inflammasome activation can also cause pyroptosis, a mechanism of cell death associated with cellular lysis and release of intracellular content into the extracellular space (Kubes et al. 2012). As touched upon above, in the absence of pathogens, DAMP-induced inflammation is termed sterile inflammation.

In the context of experiments conducted within this study, the analysis of the pro/anti-inflammatory protein secretion showed time-dependent NM-induced increases in cytokine levels (IL6 and IL8), most evident at the higher NM concentrations. Generally speaking, the cytokine secretion following exposure to the TiO_2_ and ZnO NMs was higher than the CeO_2_. Interestingly and importantly, xenobiotic-induced inflammatory response was significantly higher in the NASH MT, as compared to the steatosis MT (i.e., ten-fold increase in IL6 in NASH MT, as compared to the steatosis MT) which were again substantially higher than the healthy MT. Similarly, a concentration-dependant increase in cytokine levels was also observed in the diseased MT following exposure to Isoniazid, with the inflammatory response being significantly higher for the NASH model compared to the steatosis tissue. Once again, the data clearly shows that the disease state is absolutely vital in the governance of how the organ might deal with xenobiotic challenge, the extent of damage caused, the potential for a further inflammatory response and intensification of immune-induced organ damage.

Hepatic stellate cells are resident mesenchymal cells that retain features of both resident fibroblasts and pericytes and makeup approximately 15% of total resident cells in healthy human liver (Yin et al. 2013). These cells reside in the anti-luminal side of the fenestrated sinusoidal endothelial cell layer where exchange of biomolecules and xenobiotics occurs between portal blood flow from the gastrointestinal tract and hepatocytes. Under normal physiological conditions, quiescent HSCs are predominately involved in the storage of Vitamin A in cytoplasmic droplets. Upon activation quiescent stellate cells transdifferentiate into proliferative, migratory and contractile myofibroblasts, with distinct and potent pro-fibrogenic transcriptional and secretory properties. The activated stellate cells secrete extracellular matrix molecules that accumulate and form scar tissue (Khomich et al. 2020).

α-SMA is a reliable marker of activated and myofibroblastic hepatic stellate cells (Shang et al. 2018). A substantial body of data has shown an increase in positive immunostaining of α-SMA in specimens’ representative of progressive liver disease (i.e., Carpino et al. 2005). In this study, as anticipated higher levels of α-SMA transcripts and proteins were quantified in the NASH MTs over time as compared to the steatosis. Moreover, and more importantly, for the very first time, the data demonstrated that specific NMs were capable of activating stellate cells (most apparent for ZnO and TiO_2_ NMs). It is important to state that we do not suggest α-SMA expression to be a biomarker of NM induced hepatic damage or that the NM-induced activation of stellate cells necessarily leads to hepatic disease (as currently there is no in vivo evidence for either of these statements). Yet the data does show that NMs are capable of hepatic stellate cell activation (most important cell population in induction of hepatic fibrosis) and hence a potential for “real” hazard following repeated lifelong exposure to NMs in man cannot be ruled out.

In the final experiment, a liver-specific tissue clearance protocol was developed and utilised to allow 3D imaging of liver MT here and rodent livers (manuscripts in preparation). The autofluorescence of cleared MTs allowed for visualisation of individual cells in detail allowing for the conception of NM uptake and interaction with specific cells within the MT. The tissue clearance facilitated imaging of layers spanning the entirety of the MT allowing for a real understanding of NM distribution to the core without the necessity for cross sections. This identified certain cell populations interacted with the particulates in greater quantities as compared to the hepatocytes (believed to be non-parenchymal cells).

## Conclusion

In an effort to replace, reduce and refine the reliance on animal experimentation, there is an urgent necessity for advancing current in vitro model systems that offer features with more physiological and pathophysiological relevance and enhanced predictivity of in vivo toxicological output. Hepatic toxicology is key when considering both chemical and particulate exposure, as the liver is vital in metabolic homeostasis and detoxification of chemicals as well as being a major site of xenobiotic (in particular low solubility particulate) accumulation. An extremely crucial consideration for the liver, which has been highlighted in this study, is the importance of including representative test models from the wide-range of liver disease in risk strategies and toxicological assessment of xenobiotics. This is of significant consequence, as a large number of the general population suffer from sub-clinical liver damage without any apparent visible disease manifestations. The findings from the study highlight a number of important findings: a) establishment and validation of two disease models of the liver using a quadruple primary cell human primary MT; b) incorporation and consideration of pre-existing disease states of the liver is very important not only in the augmentation NM-induced adverse effects but also applies to pharmaceuticals and chemicals and c) NMs are able to activate hepatic stellate cells.

## Supplementary Information

Below is the link to the electronic supplementary material.

Supplementary file1 (TIF 1135 KB)

Supplementary file2 (TIF 68 KB)

Supplementary file3 (TIF 64 KB)

Supplementary file4 (TIF 181 KB)

Supplementary file5 (TIF 241 KB)

Supplementary file6 (TIF 196 KB)

Supplementary file7 (TIF 179 KB)

Supplementary file8 (TIF 156 KB)

Supplementary file9 (TIF 120 KB)

Supplementary file10 (TIF 115 KB)

Supplementary file11 (TIF 194 KB)

Supplementary file12 (PNG 14 KB)

Supplementary file13 (DOCX 24 KB)

## Data Availability

Not applicable.

## Abbreviations

α-SMA: α-Smooth muscle actin
AK: Adenylate kinase
ATP: Adenosine triphosphate
CeO_2_: Cerium dioxide
DILI: Drug-induced liver injury
DLS: Dynamic light scattering
ELISA: Enzyme-linked immunosorbent assay
HCC: Hepatocellular carcinoma
hCLS: Hepatic crown-like structures
IL: Interleukin
KC: Kupffer cells
LDL: Low-density lipoprotein
LPS: Liposaccharide
MCP-1: Monocyte chemoattractant protein-1
MIP-1α: Macrophage inflammatory protein 1α
MT: 3D microtissue
NAFLD: Non-alcoholic fatty liver disease
NASH: Non-alcoholic steatohepatitis
NM: Nanomaterials
NPC: Non-parenchymal cells
SEM: Standard error of the mean
TiO_2_: Titanium dioxide
TNF-α: Tumour necrosis factor-α
ZnO: Zinc oxide

## References

[CR1] Alam S, Mustafa G, Alam M, Ahmad N (2016). Insulin resistance in development and progression of non-alcoholic fatty liver disease. World J Gastrointestinal Pathophysiol.

[CR2] Balasubramanian SK, Jittiwat J, Manikandan J, Ong CN, Yu LE, Ong WY (2010). Biodistribution of gold nanoparticles and gene expression changes in the liver and spleen after intravenous administration in rats. Biomaterials.

[CR3] Benedict M, Zhang X (2017). Non-alcoholic fatty liver disease, an expanded review. World J Hepatol.

[CR4] Blais P, Husain N, Kramer JR, Kowalkowski M, El-Serag H, Kanwal F (2015). Nonalcoholic fatty liver disease is underrecognized in the primary care setting. Am J Gastroenterol.

[CR5] Carpino G, Morini S, Gianni Corradini S, Franchitto A, Merli M, Siciliano M, Gentili F, Onetti Muda A, Berloco P, Rossi M, Attili AF, Gaudio E (2005). Alpha-SMA expression in hepatic stellate cells and quantitative analysis of hepatic fibrosis in cirrhosis and in recurrent chronic hepatitis after liver transplantation. Dig Liver Dis.

[CR6] Du LJ, Xiang K, Liu JH, Song ZM, Liu Y, Cao A, Wang H (2018). Intestinal injury alters tissue distribution and toxicity of ZnO nanoparticles in mice. Toxicol Lett.

[CR7] Govaere O, Cockell S, Tiniakos D, Queen R, Younes R, Vacca M, Alexander L, Ravaioli F, Palmer J, Petta S, Boursier J, Rosso C, Johnson K, Wonders K, Day CP, Ekstedt M, Oresic M, Darley R, Cordell HJ, Marra F, Vodal-Puig A, Bedossa P, Schattenberg JM, Clement K, Allison M, Bugianesi E, Ratziu V, Daly AK, Anstee QM (2020). Transcriptomic profiling across the nonalcoholic fatty liver disease spectrum reveals gene signatures for steatohepatitis and fibrosis. Sci Trans Med.

[CR8] Harris R, Harman DJ, Card TR, Aithal GP, Guha IN (2017). Prevalence of clinically significant liver disease within the general population, as defined by non-invasive markers of liver fibrosis: a systematic review. The Lancet Gastroenterol Hepatol.

[CR9] Ioannou GN, Nagana Gowda GA, Djukovic D, Raftery D (2020). Distinguishing NASH histological severity using a multiplatform metabolomics approach. Metabolites.

[CR10] Itoh M, Kato H, Suganami T, Konuma K, Marumoto Y, Terai S, Sakugawa H, Kanai S, Hamaguchi M, Fukaishi T, Aoe S, Akiyoshi K, Komohara Y, Takeya M, Sakaida I, Ogawa Y (2013). Hepatic crown-like structure: a unique histological feature in non alcoholic steatohepatitis in mice and humans. PLoS ONE.

[CR11] Johnston H, Brown DM, Kermanizadeh A, Gubbins E, Stone V (2012). Investigating the relationships between nanomaterial hazard and physicochemical properties: informing the exploitation of nanomaterials within therapeutic and diagnostic applications. J Control Release.

[CR12] JRC nanomaterials repository - https://ec.europa.eu/jrc/sites/jrcsh/files/JRC-Nanomaterials-Repository-List-of-Representative-Nanomaterials.pdf (Accessed 26 May 2020).

[CR13] Kermanizadeh A, Pojana G, Gaiser BK, Birkedal R, Bilaničová D, Wallin H, Jensen KA, Sellergren B, Hutchison GR, Marcomini A, Stone V (2013). *In vitro* assessment of engineered nanomaterials using C3A cells: cytotoxicity, pro-inflammatory cytokines and function markers. Nanotoxicology.

[CR14] Kermanizadeh A, Chauché C, Balharry D, Brown DM, Kanase N, Boczkowski J, Lanone S, Stone V (2014). The role of Kupffer cells in the hepatic response to silver nanoparticles. Nanotoxicology.

[CR15] Kermanizadeh A, Balharry D, Wallin H, Loft S, Møller P (2015). Nanomaterial translocation - the biokinetics, tissue accumulation, toxicity and fate of materials in secondary organs - a review. Critical Rev Toxicol.

[CR16] Kermanizadeh A, Jacobsen NR, Roursgaard M, Loft S, Møller P (2017). Hepatic hazard assessment of silver nanoparticle exposure in healthy and chronically alcohol fed mice. Toxicol Sci.

[CR17] Kermanizadeh A, Powell L, Stone V, Møller P (2018). Nano delivery systems and stabilized solid drug nanoparticles for orally administered medicine - current landscape. Int J Nanomed.

[CR18] Kermanizadeh A, Berthing T, Guzniczak E, Wheeldon M, Whyte G, Vogel U, Moritz W, Stone V (2019). Assessment of nanoparticle-induced hepatotoxicity using a 3D human primary multi-cellular microtissue exposed repeatedly over 21 days - suitability of the in vitro test system as an in vivo surrogate. Part Fibre Toxicol.

[CR19] Kermanizadeh A, Powell LG, Stone V (2020). A review of hepatic nanotoxicology - summation of recent findings and considerations for the next generation of study designs. J Toxicol Environ Health Part B.

[CR20] Khomich O, Ivanov AV, Bartosch B (2020). Metabolic hallmarks of hepatic stellate cells in liver fibrosis. Cells.

[CR21] Kmiec Z (2010). Co-operation of liver cells in health and disease. Adv Anat Embryol Cell Biol.

[CR22] Kubes P, Mehel WZ (2012). Sterile inflammation in the liver. Gastroenterology.

[CR23] Kuna L, Bozic I, Kizivat T, Bojanic K, Mrso M, Kralj E, Smolic R, Wu GY, Smolic M (2018). Models of drug induced liver injury (DILI) - Current issues and future perspectives. Curr Drug Metab.

[CR24] Lee JH, Kim YS, Song KS, Ryu HR, Sung JH, Park HM, Song NW, Shin BS, Marshak D, Ahn K, Lee JE, Yu IJ (2013). Bio-persistence of silver nanoparticles in tissues from Sprague-Dawley rats. Part Fibre Toxicol.

[CR25] Li L, Bi Z, Hu Y, Sun L, Song Y, Chen S, Mo F, Yang J, Wei Y, Wei X (2020). Silver nanoparticles and silver ions cause inflammatory response through induction of cell necrosis and the release of mitochondria *in vivo* and *in vitro*. Cell Biol Toxicol.

[CR26] Lipka J, Semmler-Behnke M, Sperling RA, Wenk A, Takenaka S, Schleh C, Kissel T, Parak WJ, Kreyling WG (2010). Bio-distribution of PEG-Modified gold nanoparticles following intratracheal instillation and intravenous injection. Biomaterials.

[CR27] Ma B, Xu H, Zhuang W, Wang Y, Li G, Wang Y (2020). Reactive oxygen species responsive theranostic nanoplatform for two-photon aggregation-induced emission imaging and therapy of acute and chronic inflammation. ACS Nano.

[CR28] Modrzynska J, Berthing T, Ravn-Haren G, Jacobsen NR, Weydahl IK, Loeschner K, Mortensen A, Saber AT, Vogel U (2018). Primary genotoxicity in the liver following pulmonary exposure to carbon black nanoparticles in mice. Part Fibre Toxicol.

[CR29] NANOGENOTOX - https://www.anses.fr/en/system/files/nanogenotox_deliverable_5.pdf - (Accessed 24 Apr 2019).

[CR30] Safari Z, Gerard P (2019). The links between the gut microbiome and non-alcoholic fatty liver disease (NAFLD). Cell Mol Life Sci.

[CR31] Shang L, Hosseini M, Liu X, Kisseleva T, Brenner DA (2018). Human hepatic stellate cell isolation and characterization. J Gastroenterol.

[CR32] Vance ME, Kuiken T, Vejerano EP, McGinnis SP, Hochella MF, Rejeski D, Hull MS (2015). Nanotechnology in the real world: Redeveloping the nanomaterial consumer products inventory. Beilstein J Nanotechnol.

[CR33] Woolbright BL, Jaeschke H (2015). Sterile inflammation in acute liver injury: myth or mystery?. Expert Rev Gastroenterol Hepatol.

[CR34] Woolbright BL, Jaeschke H (2018). Mechanisms of inflammatory liver injury and drug-induced hepatotoxicity. Curr Pharmacol Rep.

[CR35] Yazdani HO, Chen HW, Tohme S, Tai S, Van der Windt DJ, Loughran P, Rosborough BR, Sud V, Beer-Stolz D, Turnquist HR, Tsung A, Huang H (2017). IL-33 exacerbates liver sterile inflammation by amplifying neutrophil extracellular trap formation. J Hepatol.

[CR36] Yin C, Evason KJ, Asahina K, Stainier DYR (2013). Hepatic stellate cells in liver development, regeneration, and cancer. J Clin Investig.

[CR37] Younossi Z, Anstee QM, Marietti M, Hardy T, Henry L, Eslam M, George J, Bugianesi E (2018). Global burden of NAFLD and NASH: trends, predictions, risk factors and prevention. Nat Rev Gastroenterol Hepatol.

