# Supplementary figures and images for: Particulate and drug-induced toxicity assessed in novel quadruple cell human primary hepatic disease models of steatosis and pre-fibrotic NASH
Source: Arch Toxicol. 2021 Oct 20;96(1):287–303. doi: 10.1007/s00204-021-03181-2 (PMC8748349; doi:10.1007/s00204-021-03181-2)

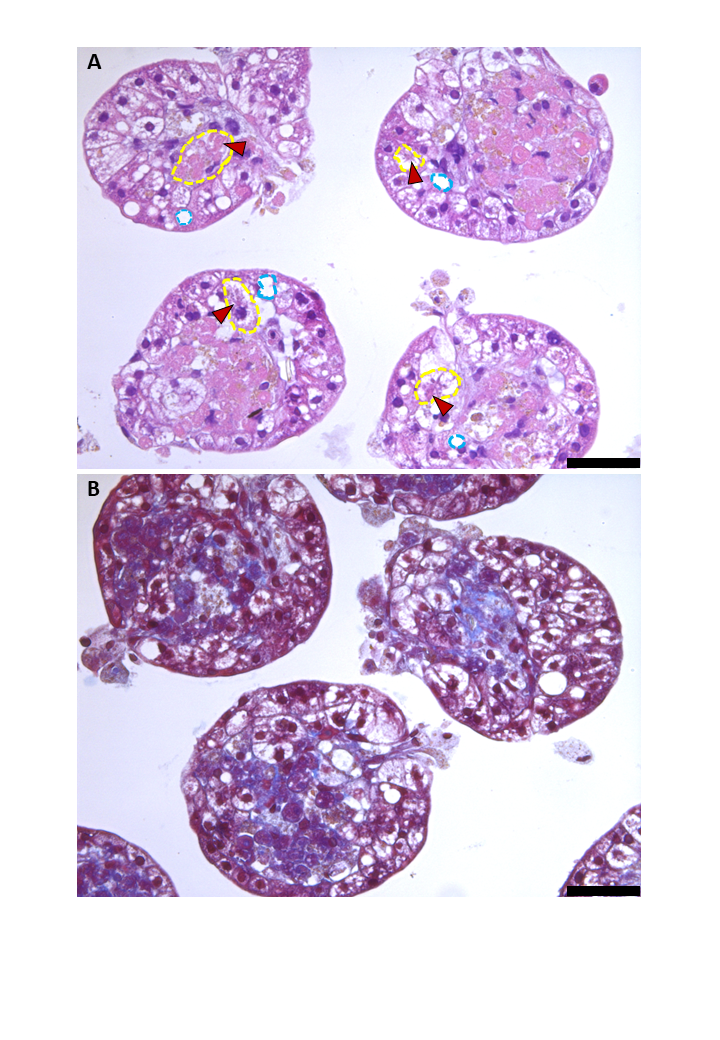

Supplement: Supplementary file 1 — Supplementary file1 (TIF 1135 KB) [file 204_2021_3181_MOESM1_ESM.tif]

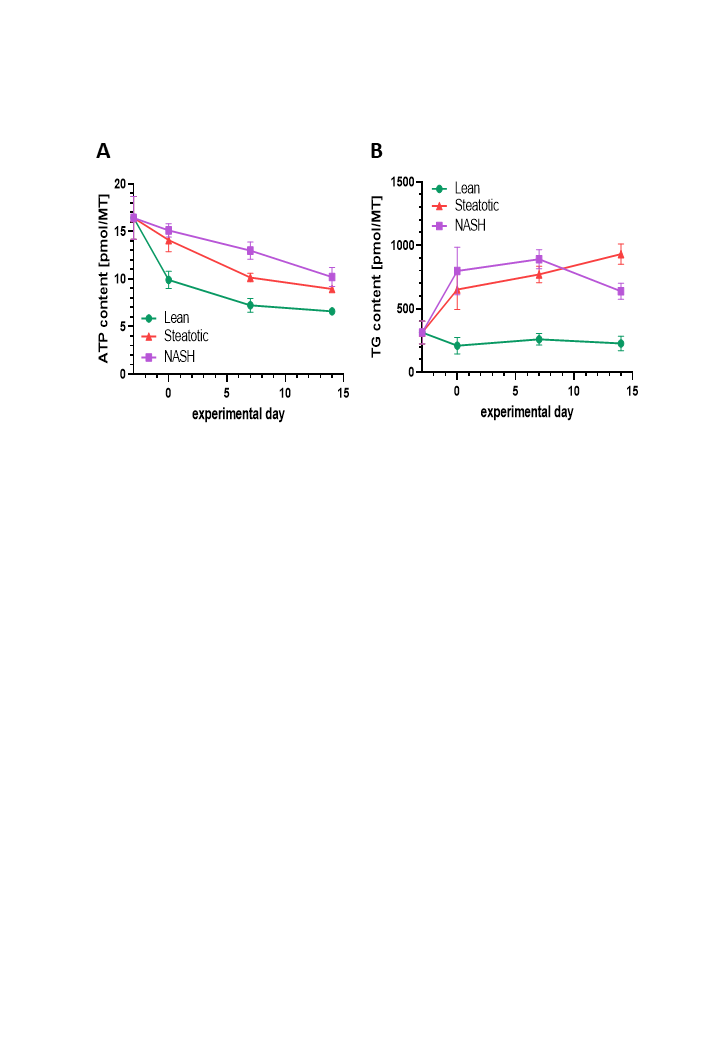

Supplement: Supplementary file 2 — Supplementary file2 (TIF 68 KB) [file 204_2021_3181_MOESM2_ESM.tif]

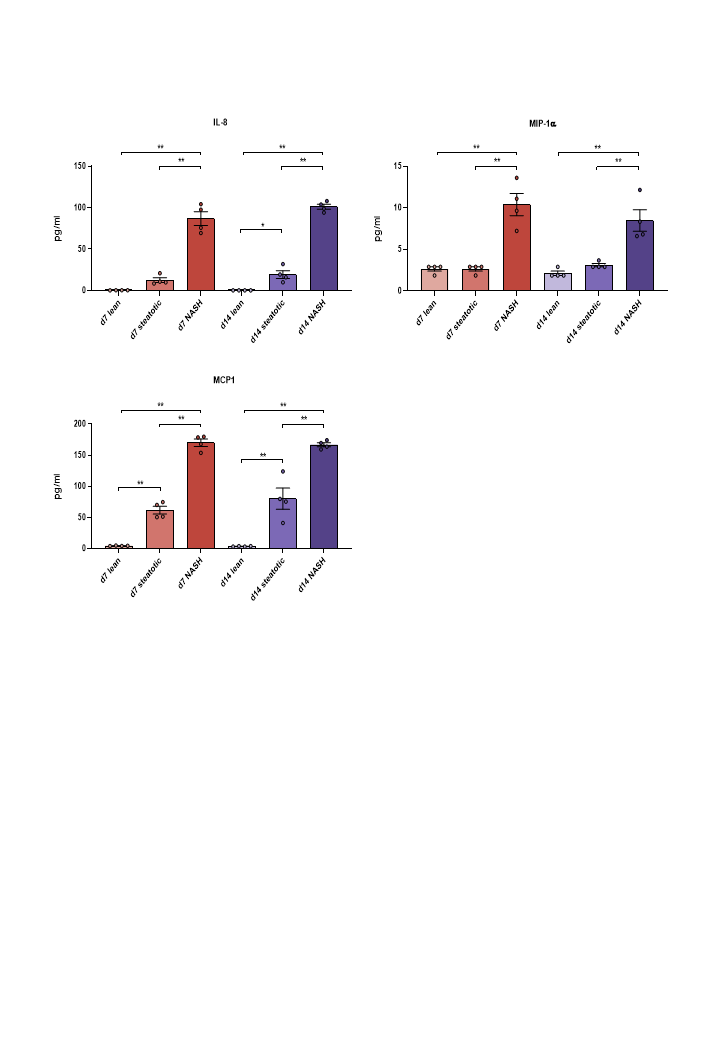

Supplement: Supplementary file 3 — Supplementary file3 (TIF 64 KB) [file 204_2021_3181_MOESM3_ESM.tif]

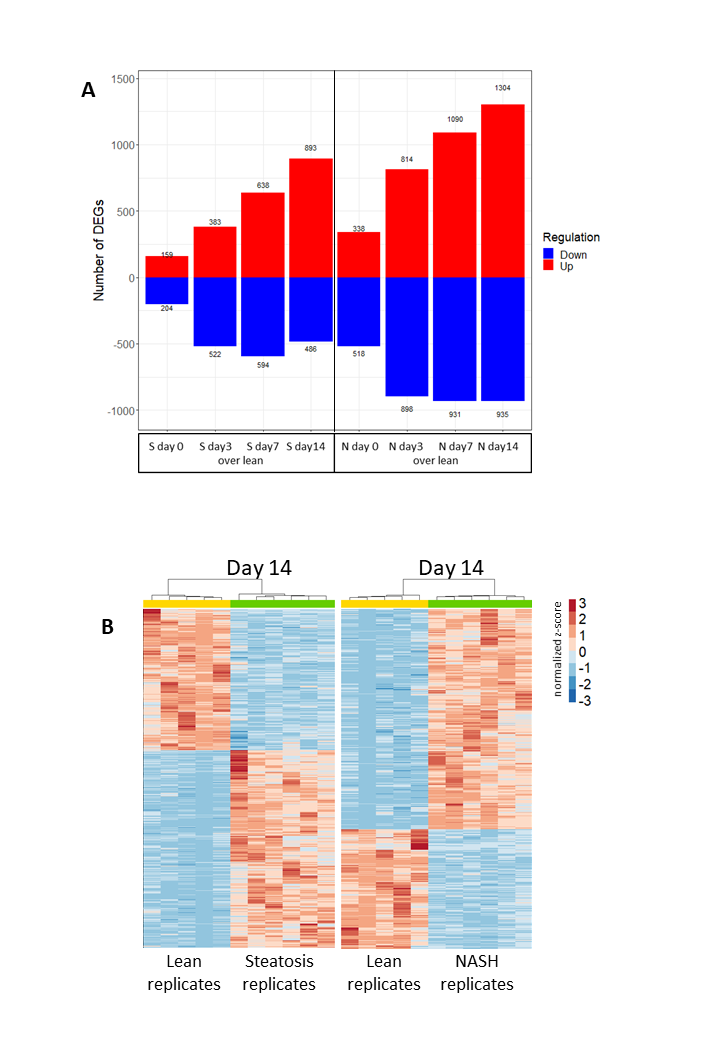

Supplement: Supplementary file 4 — Supplementary file4 (TIF 181 KB) [file 204_2021_3181_MOESM4_ESM.tif]

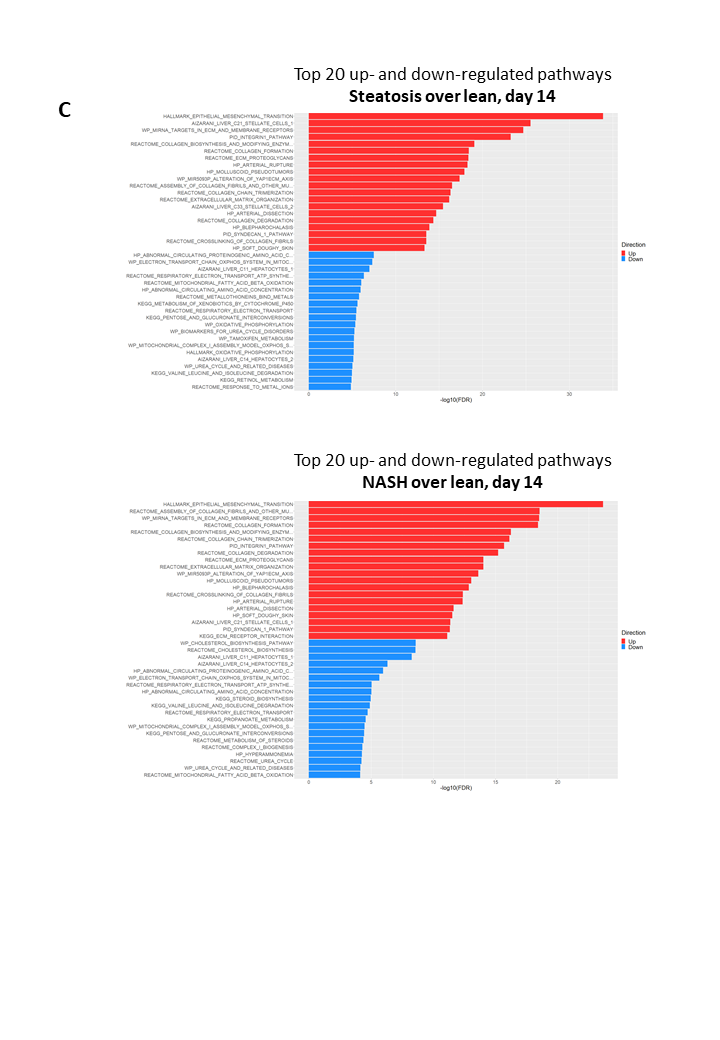

Supplement: Supplementary file 5 — Supplementary file5 (TIF 241 KB) [file 204_2021_3181_MOESM5_ESM.tif]

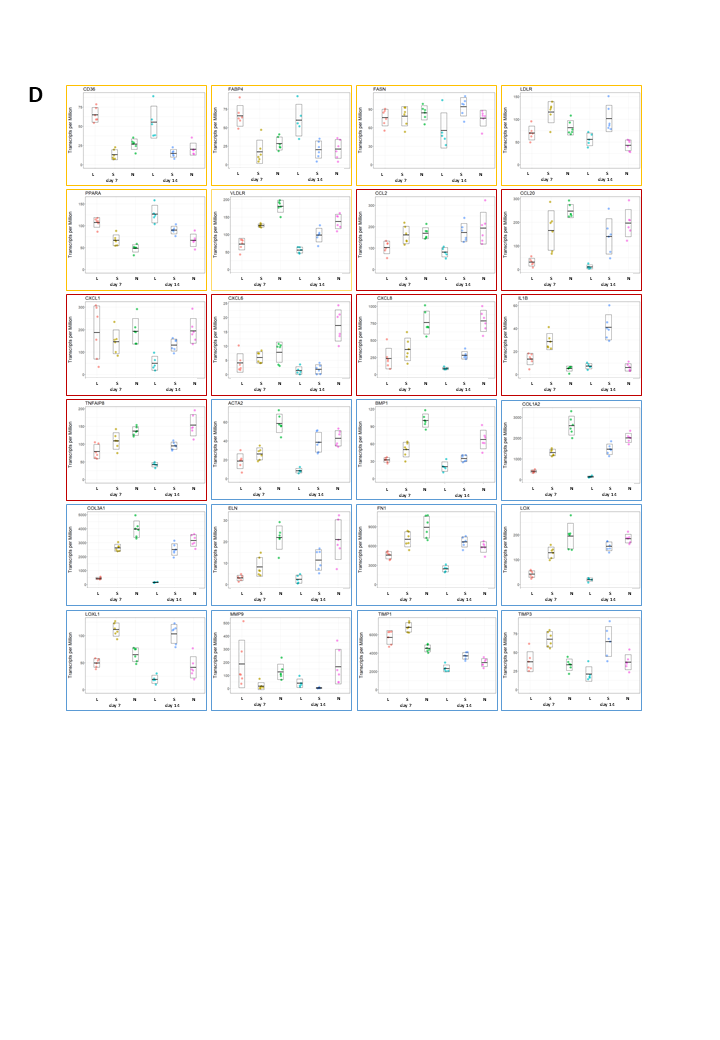

Supplement: Supplementary file 6 — Supplementary file6 (TIF 196 KB) [file 204_2021_3181_MOESM6_ESM.tif]

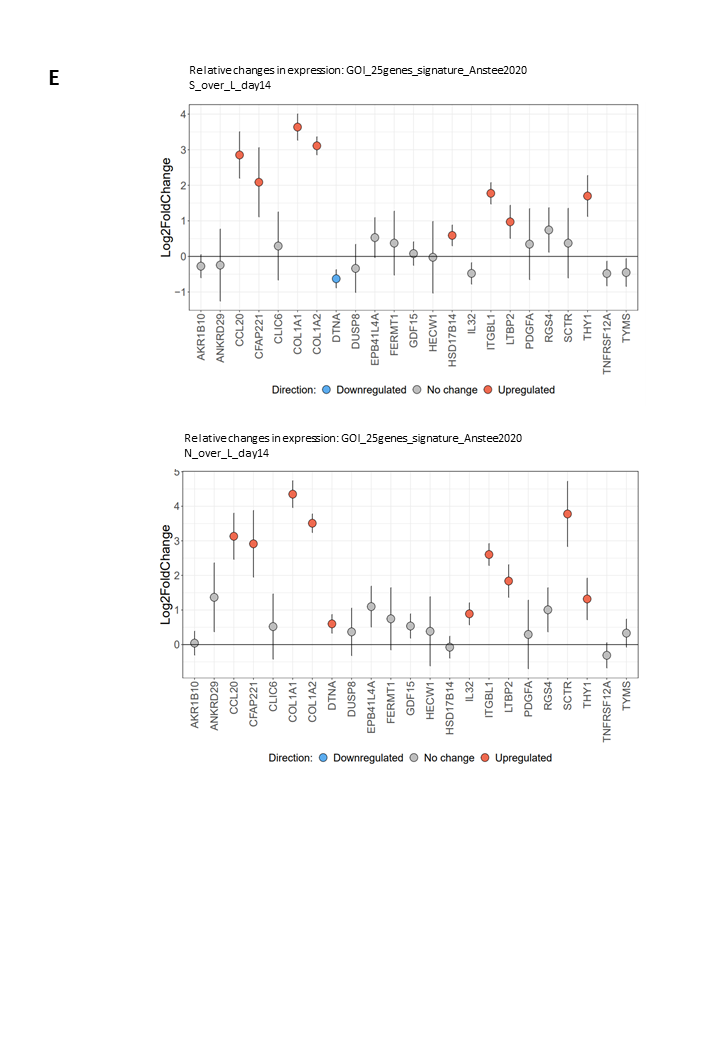

Supplement: Supplementary file 7 — Supplementary file7 (TIF 179 KB) [file 204_2021_3181_MOESM7_ESM.tif]

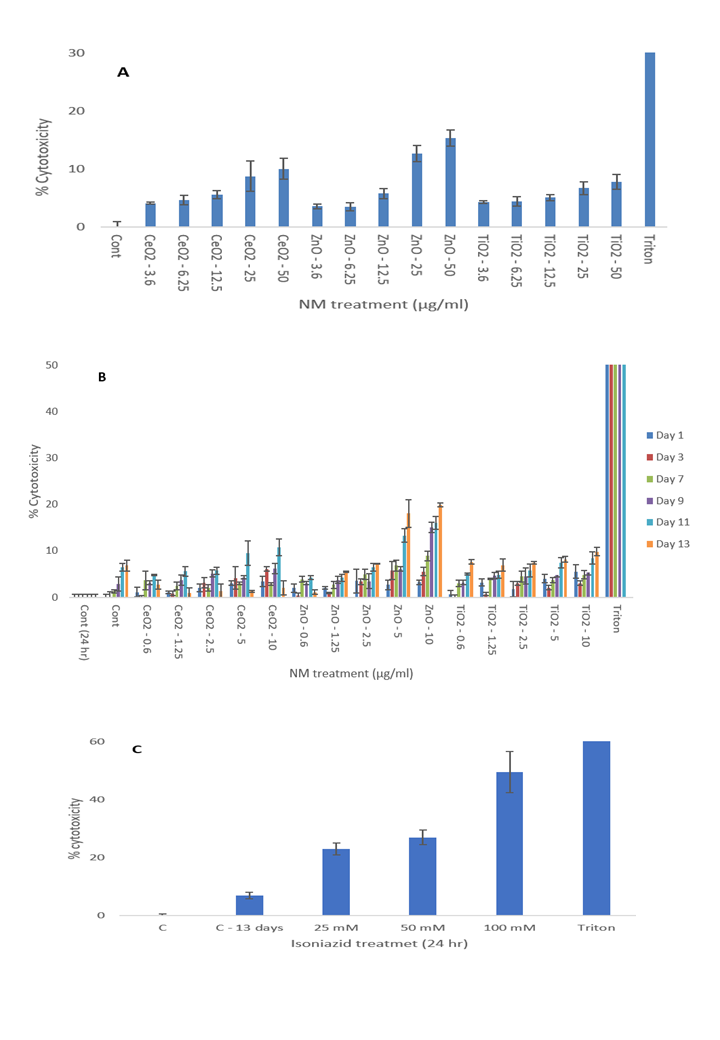

Supplement: Supplementary file 8 — Supplementary file8 (TIF 156 KB) [file 204_2021_3181_MOESM8_ESM.tif]

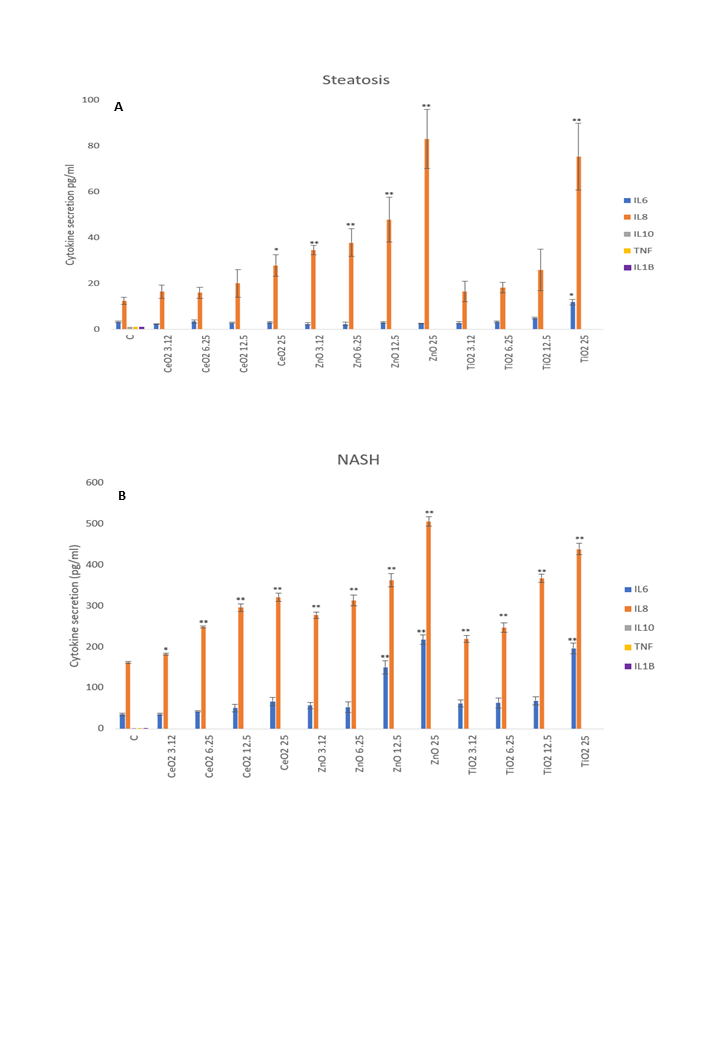

Supplement: Supplementary file 9 — Supplementary file9 (TIF 120 KB) [file 204_2021_3181_MOESM9_ESM.tif]

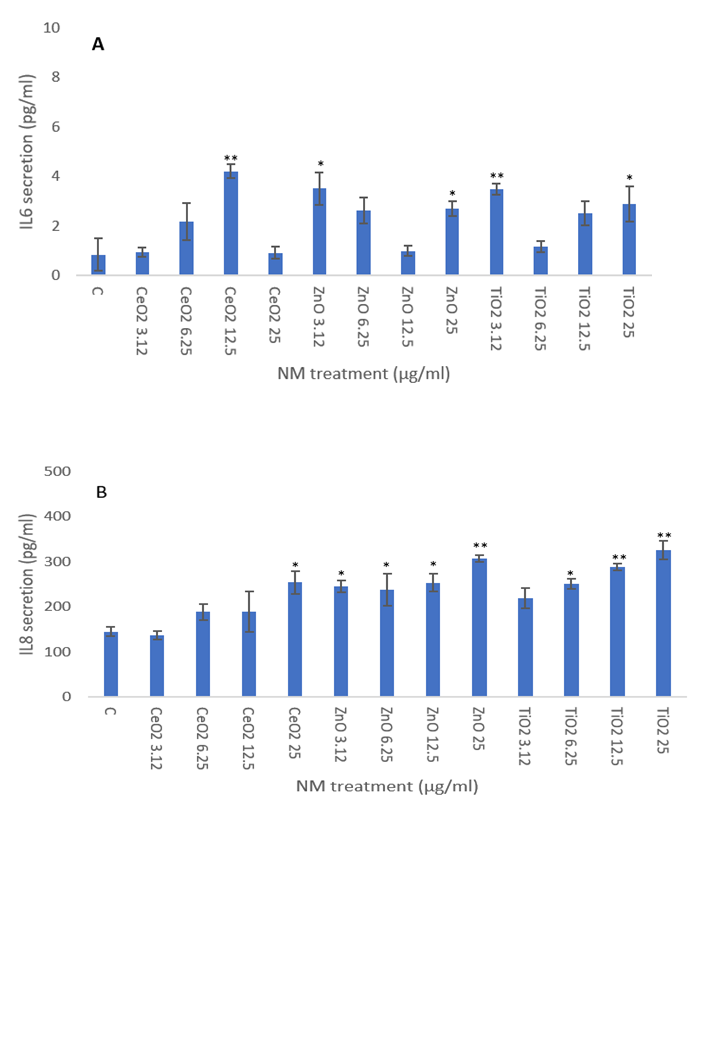

Supplement: Supplementary file 10 — Supplementary file10 (TIF 115 KB) [file 204_2021_3181_MOESM10_ESM.tif]

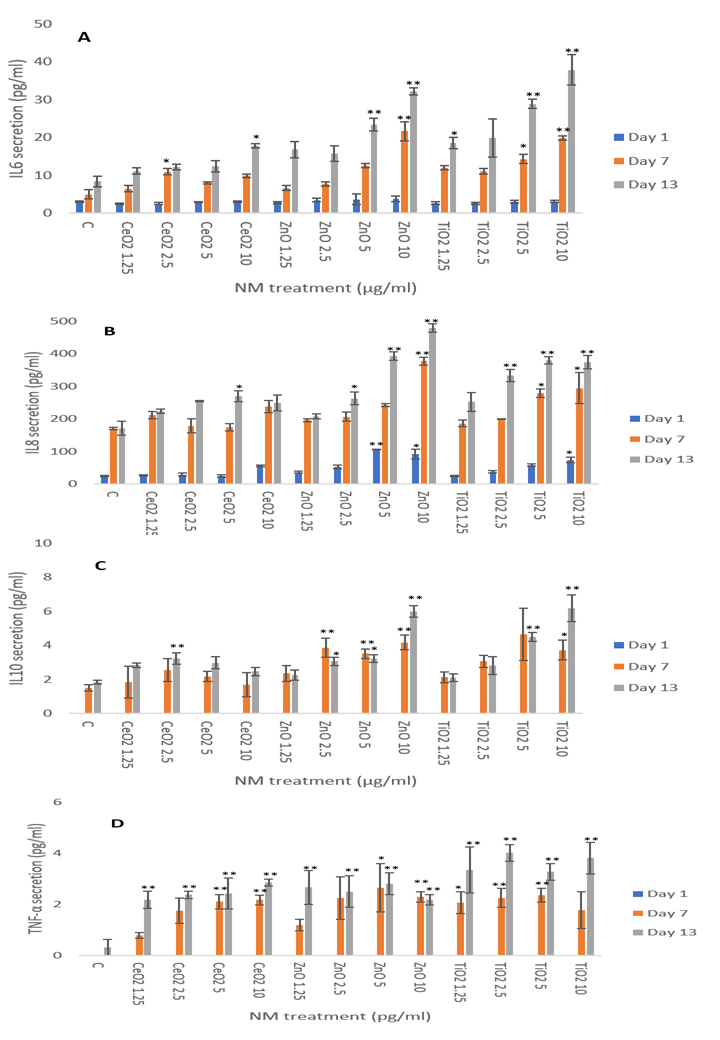

Supplement: Supplementary file 11 — Supplementary file11 (TIF 194 KB) [file 204_2021_3181_MOESM11_ESM.tif]

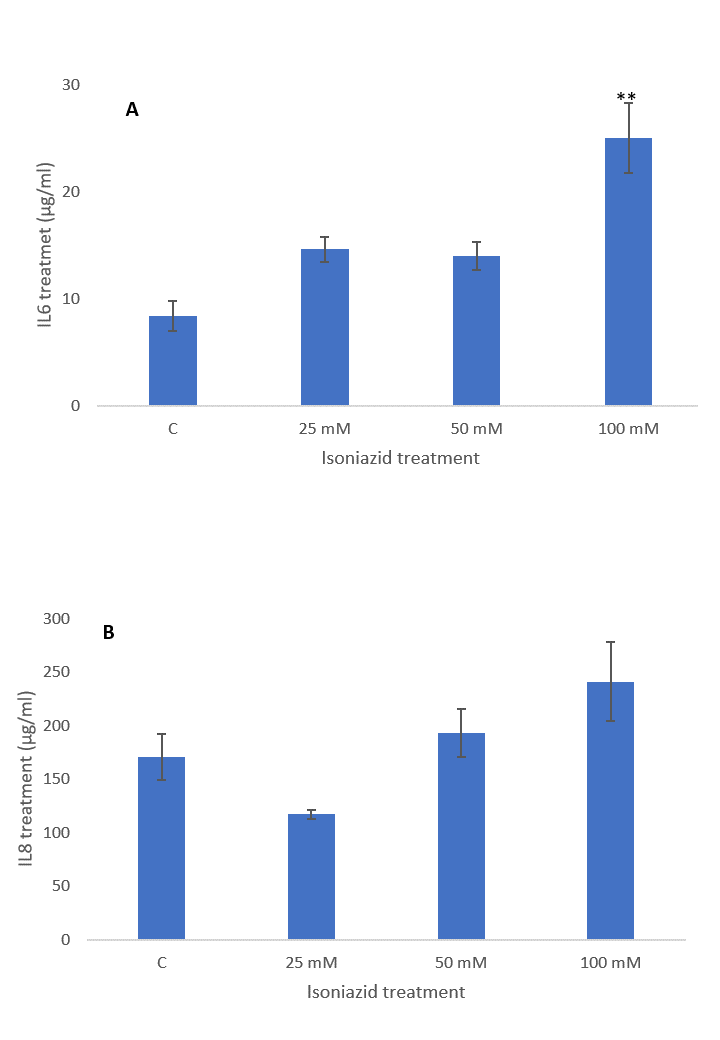

Supplement: Supplementary file 12 — Supplementary file12 (PNG 14 KB) [file 204_2021_3181_MOESM12_ESM.png]
