# Supplementary material for: Particulate and drug-induced toxicity assessed in novel quadruple cell human primary hepatic disease models of steatosis and pre-fibrotic NASH
Source: Arch Toxicol. 2021 Oct 20;96(1):287–303. doi: 10.1007/s00204-021-03181-2 (PMC8748349; doi:10.1007/s00204-021-03181-2)
Supplement: Supplementary file 13 — Supplementary file13 (DOCX 24 KB) [file 204_2021_3181_MOESM13_ESM.docx]

**Table 1.** Main physical and chemical properties of investigated materials (adapted and reproduced from JRC nanomaterials repository 2020; Kermanizadeh et al. 2013; 2019). The hydrodynamic size distributions of the NMs dispersed in complete cell culture medium were determined at a concentration of 10 µg/ml by Dynamic Light Scattering (DLS).

| **Material code** | **Material type** | **Phase** | **Primary size (nm)** | **Surface area**  **[m^2^/g]**  **(BET)** | **Known coating** | **Size in liver maintenance medium (DLS) (nm)^Ψ^** |
| --- | --- | --- | --- | --- | --- | --- |
| JRCNM01101a | ZnO | Coated | 152 | 15 | \| Triethoxy-  caprylylsilane \| \| --- \| | 692.2±78.5 |
| NM212 | CeO_2_ | Irregular and non-homogeneous | 49 | - | None | 512.9±8.2 |
| E171 | Food grade TiO_2_ | 99.8% anatase and 0.2% rutile | - | - | None | 614±5.6 |

^Ψ^ Size in biological media measured within 30 min of sonication.

**Table 2.** Time-line for toxicological end-points measured in the single or repeated exposure experiments over a period of 2 weeks

| **Experiment 1 and 2 and 9** | **End-points investigated** |
| --- | --- |
| Day 1 | **AK assay,** **cytokine secretion,** **α-SMA** |

| **Experiment 3 and 4** | **End-points investigated** |
| --- | --- |
| Day 1 | **AK assay, cytokine secretion** |
| Day 3 | **AK assay,** |
| Day 5 | **AK assay** |
| Day 7 | **AK assay** |
| Day 13 | **AK assay, cytokine secretion** |

| **Experiment 5, 6 and 10** | **End-points investigated** |
| --- | --- |
| Day 1 | **AK assay,** **cytokine secretion** |
| Day 3 | **AK assay,** |
| Day 5 | **AK assay** |
| Day 7 | **AK assay** |
| Day 9 | **AK assay, cytokine secretion** |
| Day 11 | **AK assay** |
| Day 13 | **AK assay, cytokine secretion, α-SMA** |

| **Experiment 7, 8 and 11** | **End-points investigated** |
| --- | --- |
| Day 13 | **AK assay,** **cytokine secretion** |

**Table 3.** Tissue clearance protocol for primary human liver MT

| **Reagent** | **Incubation time** |
| --- | --- |
| 50% tetrahydrofuran (THF) (vol/vol) | 25 mins |
| 70% tetrahydrofuran (THF) (vol/vol) | 25 mins |
| 80% tetrahydrofuran (THF) (vol/vol) | 25 mins |
| 100% tetrahydrofuran (THF) (vol/vol) | 3 x 25 mins |
| 100% dichloromethane | 20 mins |
| 100% dibasic ether | 15 mins |
